# Uncontacted Waorani in the Yasuní Biosphere Reserve: Geographical Validation of the *Zona Intangible Tagaeri Taromenane (ZITT)*

**DOI:** 10.1371/journal.pone.0066293

**Published:** 2013-06-19

**Authors:** Salvatore Eugenio Pappalardo, Massimo De Marchi, Francesco Ferrarese

**Affiliations:** 1 Department of Agronomy, Food, Natural Resources, Animals and the Environment, University of Padova, Padova, Italy; 2 Department of Civil, Environmental Architectural Engineering, University of Padova, Padova, Italy; 3 Prometeo Project, Universidad Nacional de Chimborazo, Riobamba, Ecuador; 4 GIS Laboratory, Department of Historical, Geographical and Antiquity Sciences, University of Padova, Padova, Italy; University of Utah, United States of America

## Abstract

The Tagaeri Taromenane People are two indigenous groups belonging to the Waorani first nation living in voluntary isolation within the Napo region of the western Amazon rainforest. To protect their territory the Ecuadorean State has declared and geographically defined, by Decrees, the *Zona Intangible Tagaeri Taromenane* (ZITT). This zone is located within the UNESCO Yasuní Biosphere Reserve (1989), one of the most biodiverse areas in the world. Due to several hydrocarbon reserve exploitation projects running in the area and the advancing of a large-scale deforestation front, the survival of these groups is presently at risk. The general aim was to validate the ZITT boundary using the geographical references included in the Decree 2187 (2007) by analyzing the geomorphological characteristics of the area. Remote sensing data such as Digital Elevation Models (DEM), Landsat imagery, topographic cartography of IGM-Ecuador, and fieldwork geographical data have been integrated and processed by Geographical Information System (GIS). The ZITT presents two levels of geographic inconsistencies. The first dimension is about the serious cartographical weaknesses in the perimeter delimitation related to the impossibility of linking two rivers belonging to different basins while the second deals with the perimeter line not respecting the hydrographic network. The GIS analysis results clearly show that ZITT boundary is cartographically nonsense due to the impossibility of mapping out the perimeter. Furthermore, GIS analysis of anthropological data shows presence of Tagaeri Taromenane clans outside the ZITT perimeter, within oil production areas and in nearby farmer settlements, reflecting the limits of protection policies for non-contacted indigenous territory. The delimitation of the ZITT followed a traditional pattern of geometric boundary not taking into account the nomadic characteristic of Tagaeri Taromenane: it is necessary to adopt geographical approaches to recognize the indigenous right to their liveable territories in the complex territorialities enacted by different stakeholders.

## Introduction

The Tagaeri Taromenane are two indigenous groups living in “Voluntary Isolation” in the Napo Moist terrestrial ecoregion, located in the western Amazon rainforest [1]–[5]. This ecoregion is one of the most biologically and culturally diverse areas on the planet [6], [7] representing an extraordinary richness across several taxa (amphibian, mammal, bird and plants), a high level of regional endemism [8]–[10], and is home to several indigenous ethnic groups including some of the world's last uncontacted peoples [11].

The Tagaeri Taromenane are settled in the Ecuadorean Amazon Region between the Yasuní and Curaray rivers and within the ancestral territory of the Waorani (or Huaorani) indigenous first nation [12], [13].

To protect the territory of these uncontacted indigenous people in the Yasuní Biosphere Reserve, the national government declared (1999) and mapped (2007), through two Presidential Decrees, the “No-Go-Zone” called Intangible Zone or *Zona Intangible Tagaeri Taromenane* (ZITT) [14], [15].

Decree 2187 of 2007 represents the first official text that uniquely gives, using a geographical language, spatially explicit information to define the perimeter of ZITT.

**Figure 1:**
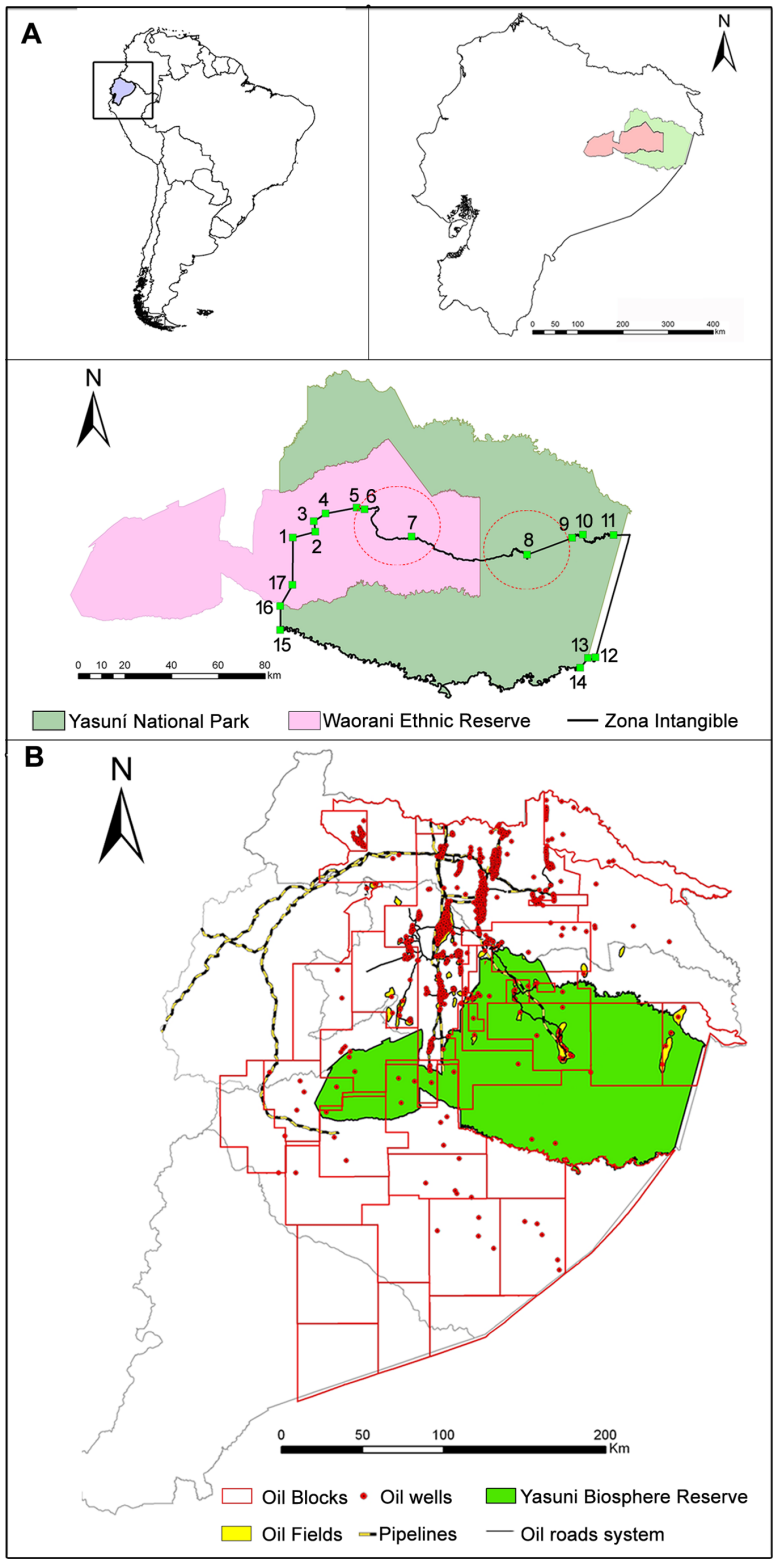
Yasuní Biosphere Reserve and *Zona Intangible Tagaeri Taromenane* (ZITT): geographical framework. A) Delimitation of the *Zona Intangible* by given geographical coordinates and critical hotspots (red circles); B) Oil production in the Ecuadorian Amazon Region (9^th^ concession licensing round, 2001).

The boundary of the ZITT is clamped onto 17 univocal points geographically expressed by pairs of metrical coordinates declared in the Decree 2187 (Figure 1A). To map the borders of the ZITT it is required to join the pairs of coordinates by rectilinear segments and by sections of river courses according to the geomorphological descriptions included in the Decree 2187.

Due to different interpretations of the geographical references expressed in the Decree 2187 and to the complex geomorphological characteristics of the Amazon sub-basins, different map representations of the ZITT are demarcated both in the official and unofficial cartography of the Yasuní Biosphere Reserve [16]–[20].

Several studies about the ZITT and the issue on the uncontacted indigenous groups have been produced using mainly social and anthropological approaches [2], [3], [12], [13], [21]–[25]. No geographical studies and GIS spatial analysis on the ZITT have been produced in the scientific literature.

Fieldwork activities in the Yasuní Biosphere Reserve, geographical analysis of the Decree 2187, and studies on geomorphological characteristics of the area suggested the hypothesis that the official boundary of the ZITT is cartographically inaccurate with serious geographical inconsistencies along the perimeter.

Crucial geographical inconsistencies seem localized between points No. 6 and No. 7 and between No.7 and No. 8.

The general aim of this paper is to validate the boundary using the geographical references expressed in the Decree 2187 of 2007 by analyzing the geomorphological characteristics of the area in order to re-map the perimeter of the ZITT through spatial and remote sensing analysis.

The GIS analysis results clearly show geographical incoherencies in two hotspots of the Yasuní and unequivocally demonstrate the impossibility to map the perimeter of the ZITT.

Analyses of both anthropological geospatial data about the Tagaeri Taromenane and their spatial relationships with extractive activities and roads confirm the existence of uncontacted groups in a wider area crossing oil fields and farmer settlements outside the same boundary of the *Zona Intangible*.

Furthermore, violent contacts between Tagaeri Taromenane group and external *mestizo* farmers within the territory district of Dayuma (close to the Hormiguero extractive platform and the Armadillo oil field in 2009) [26]–[28] and maybe with Waorani relative (in 2013 within oil block 16, near the Maxus Road) happened in the area of Yasuní. Between the 4^th^ and 5^th^ of March 2013 two old Waorani were speared to death. The attack is under investigation by Ecuadorian Authorities to confirm responsibility of Tagaeri Taromenane groups [29]. These facts confirmed once more the presence of the Tagaeri Taromenane also outside the ZITT perimeter (Figures S1, S2), fuelling a hot debate inside national society questioning the relationship among the majority and minorities and asking for a different way of conceiving territories: from the Westphalian rigid geometry to a geographic network of cultural and environmental relationships.

### The overlapping systems of territorial complexities

Through the Man and Biosphere Program, the United Nation's Agency UNESCO included this geographical area into the Biosphere Reserve network (1989), declaring the Yasuní National Park and the land-titled of Waorani territory (known as Waorani Ethnic Reserve) as Yasuní Biosphere Reserve. It was added in order to prioritize and to conciliate biodiversity conservation with sustainable development in the territory planning [30], [31].

However, due to huge non-renewable energy reserves and to the crucial role they play in the national economy [32]–[34], the Ecuadorean State zoned specific geographical areas in the Amazon Region for hydrocarbon industrial activities – the so called “oil blocks” – so that, according to the 9th licensing round established in 2001, roughly the 79% of the YBR [35]–[37] was overlapped by concessions for oil extraction and production (Figure 1B) [38].

Oil development in the Ecuadorian Amazon (Figure 1B) has already directly and indirectly caused major social and environmental change to the Amazon people and ecosystems. Direct effects include deforestation for drilling platforms, pipelines, access roads, seismic prospecting activities, and chemical contamination of water bodies from wastewater discharges, oil spills and gas emissions [39]–[43]. Indirect effects are related to the opening of roads for oil exploration and transportation which turns terrestrial communications infrastructures into the main vector for colonization of primary forest [44]–[46], represented by the Moist Tropical Forest. Colonization of Moist Tropical Forest is causing increased deforestation as well as logging and hunting from human settlements [47]–[50]. As it is interpreted by the satellite image of the Via Auca Road (Landsat ETM+, 2002; Figure S1) – the most important oil terrestrial infrastructure at the western sector of the Yasuní Biosphere Reserve – colonization processes of the forests are usually driven by the main road axis [51]. They represent the main driver of extensive deforestation activity traced out by orthogonal and parallel processes resulting in land cover/land use change dynamics in the typical deforestation spatial pattern called fish-bone (Figure S1) [52]–[56]. Moreover, rapid expansion of Via Auca road system to the west underlies direct anthropogenic pressure to Tagaeri Taromenane mobility space [38]. Previous GIS analysis by processing GPS data of the Via Auca road network showed, between 2009 and 2012, a spatial evolution to the west of 100 linear km (Figure S2) [38], [57]. Land use and land cover change dynamics result in agricultural activities both at intensive cultivation level (i.e. African Palm, *Elaeis guineensis* bot. sp.) and non-traditional farming (Figures S1, S2).

Therefore, hydrocarbon reserves exploitation, by its direct and indirect impacts on tropical forest ecosystems, played a pivotal role in turning the Napo ecoregion into one of the 14 mayor deforestation fronts in the world [58]–[60].

This set of socio-environmental processes taking place in the Ecuadorian Amazon (especially in the Auca territory system) is also affecting the social reproduction and the same survival of the Tagaeri Taromenane, clans of uncontacted hunters, and collectors living by semi-nomadic lifestyle in a wide territory of the Yasuní Biosphere Reserve's western and southern sector [3], [13], [61].

Additionally, in the last decades, several violent contacts between uncontacted indigenous people and external stakeholders induced the Inter-American Commission on Human Rights (IACHR, 2006) to grant precautionary measures in favor of the Tagaeri Taromenane demanding that “the Ecuadorian State adopt measures necessary to protect the territory inhabited by beneficiaries from third parties” [62].

The institutional history of the Intangible Zones began in the 1999 by the Decree 552, declaring the *Zona Intangible* to protect the territory of the uncontacted Tagaeri Taromenane indigenous groups. This Decree stated to identify and geographically define the ZITT within a time range of 120 days from the 2^nd^ of February 1999 [14]. After 4 years, by the Inter-ministerial Agreement 092, a Technical Commission was saddled with establishing the perimeter of the ZITT (Quito, 2004) along with the task of identifying criteria including the integration of monitoring and controlling systems of the area [63]. The delimitation of the ZITT has been a long and controversial process [2], [64] complicated by the fact that this area is “geographically embedded” within the industrial concession areas for oil exploitation [65]. On the north and west sides, there are oil companies operating in industrial production for the past three decades while on the south side there are the exploratory blocks which represent, by the 11^th^ licensing round opened in November 2012 [66], a spatial shift to the Southern Amazonian sector of the extractive frontier (Figure 1B) [67]–[70].

Finally, a geographic delimitation of the ZITT was issued on the 16^th^ of January 2007 through the Decree 2187 signed by the President of the Republic Alfredo Palacio [15]. To mitigate external anthropic influences, a buffer zone of 10 km has been defined around its perimeter. Within this additional protected area, wood extraction activities and new oil concessions are forbidden, while traditional activities such as hunting, fishing, and traditional use of biodiversity is allowed to the ancestral indigenous communities (the Waorani first nation) (Art. 2, Decree 2187) [15].

According to the Ecuadorean Decree the Intangible Zones are defined as “protected areas of high cultural and biological importance in which whatever extractive activities are forbidden due to its high value for the Amazon rainforest, Ecuador, the World and the present and future generations” (1999, Presidencia de la Repubblica) [71]. It is therefore an “untouchable zone” in which oil, gas, and other external anthropic activities must be off limits.

The ZITT is also a crucial issue because it is related to the “Yasuní-ITT initiative” undertaken by the Ecuadorean Government (2007) [72]–[74], an international initiative oriented to protect biodiversity, recognize indigenous peoples' territory, and contrast climate change. The “Yasuní-ITT Initiative” proposal aims to keep locked underground, in perpetuity, at least 850 million barrels of heavy crude oil. This will prevent the emission of CO_2_ associated with burning fossil fuels of about 410 million metric tons of out of the atmosphere, in exchange of financial compensation from the developed countries concerned about tropical deforestation and climate change [75], [76].

## Results and Discussion

### Cartographic analysis of the 17 vertex coordinates

The boundary of the ZITT is cartographically defined by Art. 1 of Decree 2187. The perimeter is spatially determined by 17 given points expressed by metric coordinates (UTM projection, zone 18, PSAD1956 geographic projection system) which have been cartographically validated. They are all geographically plausible and do not have any transcription errors. On the one side, all of the 17 vertex coordinates are consistent both with the geomorphological description and the measurements of straight lines included in the text of Decree 2187; however, on the other side sections of river courses linking vertex coordinates are in some segments ambiguous. In the latter case, mapping out the boundary becomes more complicated.

Marking out river segments of the ZITT boundary imply the use large scale maps (1:50,000 or 1:25,000), including a detailed representation of hydrographic networks, the relief system, and toponyms. Due to a complex and vast territory characterized by the typical Moist Tropical Forest land cover and by a hydrographic system evolving into a very dense network, geographic interpretation could be difficult for cartographers since it is easily prone to cartographic errors.

**Figure 2:**
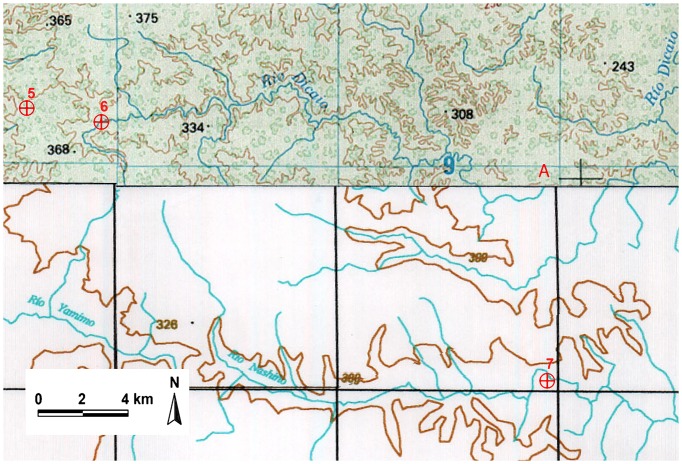
Perimeter of *Zona Intangible Tagaeri Taromenane* (ZITT) including points No. 5, No. 6, No. 7. (IGM Ecuador, scale 1:250,000). Points No. 6 and No. 7 belong to different rivers: the former to the Rio Dicaro, the latter to the Rio Nashiño.

**Figure 3:**
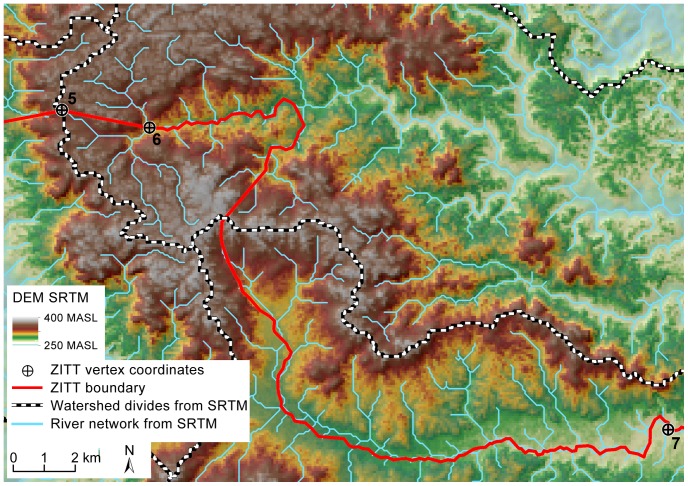
Digital Elevation Model (SRTM) in the area of points No. 5 and 6. DEM analysis of river basins and catchment's divides: Rio Dicaro and Rio Nashiño belong to different basins: the first one flows into the Yasuní River, the second one into the Curaray River. Therefore “following downstream the Rio Dicaro till point No. 7 by the Rio Nashiño” (Art. 1, indent 3) [15] becomes a “geographical nonsense” in mapping out the perimeter section from points No. 6 to that No. 7.

Cartographic delimitation of the ZITT starts from point No. 1 which reaches by a straight line point No. 2. From here the boundary overlaps the Cononaco Chico River to the north until point No. 3. This segment of the limit is short (4,550 meters) and it does not create any problems of interpretation due to the importance of this river which is well represented in the cartography production of IGM. From point No. 3 to No. 4, the boundary is marked out by a straight line of 6,140 meters according to the given coordinates. The border continues from point No. 4 to No. 5 again by a straight line. From point No. 5 to No. 6 the perimeter line follows once again a straight line “till reaching the confluence of two tributaries of the Rio Bahameno in point No. 6, having 9892355 North and 339450 Este” (2^nd^ indent, Art. 1, Decree. 2187) (Document S1). We noticed that, although the linear distance between these points is consistent to the values of the given coordinates (Table S1) and point No. 6 exactly lies on a river confluence, the two tributaries do not belong to the Rio Bahameno but to the Rio Dicaro (Figure 2, 3). As a matter of fact, it is the same geomorphological description in the official text stating that from point No. 6 the boundary follows the Rio Dicaro downstream in the east direction.

The most controversial part has been identified in the perimeter section between point No. 6 and No. 7: “(the boundary) follows to East direction by the Rio Dicaro course, then it runs downstream by the Rio Nashiño till reaching point No. 7” (3^rd^ indent, Decree 2187) (Document S1) [15]. As mentioned above, the ZITT limit runs downstream along the Rio Dicaro in the east direction. Once proved that point No. 6 matches up with the riverbed of the Rio Dicaro (not the Rio Bahameno) and point No. 7 properly overlies the Rio Bahameno, it is important to understand how it has been operated the leap of river basins. It is clear that the Rio Dicaro and Rio Nashiño belong to two different river basins. The first one flows into the Rio Yasuní and the second into the Rio Curaray.

Assuming the linear distance between point No. 6 and No. 7 is about 23,300 meters (Table S1), this particular step of the Decree 2187 technically gives infinite solutions. Therefore, if there is no better explanation, this perimeter section becomes cartographically unresolved. In any case, it is not possible to follow downstream the Rio Dicaro until point No. 7. This discrepancy results in geomorphological inconsistencies if it is not made explicit where to leave the Rio Dicaro course.

From point No. 7 to No. 8 the boundary follows the river course of Rio Nashiño. The distance corresponds to more than 50 km (Table S1). From this point the limit continues until point No. 9 by a straight line approximately 20.6 km to the north-east direction, according to the values of coordinates declared in the Decree 2187.

From point No. 9 to No. 10 the ZITT perimeter follows the river course of Rio Yasuní and from that point by a straight line of 6,850 meters reaches point No. 11. This point coincides to the same state border between Ecuador and Peru.

From point No. 11 to that No. 12 the boundary is traced out by a straight line of 53,978 meters oriented in the south-west direction. The given coordinates of point No. 12 matches the river course of Rio Cononaco which has to be followed upstream to point No. 13. From here, by a straight line of 5,438 meters, the ZITT limit reach point No. 14 which overlaps the course of Rio Curaray. From this point the perimeter section follows the Curaray River upstream in the west direction until point No. 15. From point No. 15 to No. 16 the boundary is trace out by a straight line 10,190 meters long orientated in the north direction. It then heads north-east by another straight segment of 10,500 meters to reach point No. 17. From this point to that No. 1 the limit line close the perimeter by another straight line headed north.

According to geographic interpretation of spatially explicit data contained in Art. 1 of the Decree 2187, the ZITT perimeter from point No. 7 to No. 17 does not show cartographic inconsistencies nor geomorphological ones.

### Rio Dicaro and Rio Nashiño: the river basins analysis

Basins and river dynamics analyses have been carried out to clarify the most critical perimeter section of the ZITT expressed between points No. 6 and No. 7.

The GIS analysis is based on comparing three different Digital Elevation Models (DEM) that clearly represent the terrain morphology: an IGM DEM map [77], an Aster GDEM (second version) map [78], and a SRTM map [79].

The DEM analysis of river basins spatial relationships clearly show the catchment's divides of the Rio Dicaro and the Rio Nashiño river basins. Using the results from three different DEM, maps demonstrate that it is not possible to operate a leap of basin to link out points No. 6 to that No. 7 (Figures 3, S3); however, according to the Decree 2187 text, it is clear that somewhere this leap has to be done.

Therefore, “following downstream the Rio Dicaro till point No. 7 by the Rio Nashiño” (Art. 1, indent 3, Decree 2187) (Document S1) [15] becomes “geographical nonsense” in mapping out the perimeter section from points No. 6 to that No. 7. Due to this geographic ambiguity, different representations of the ZITT boundary in this area have been mapped out in official and unofficial cartography production on the Yasuní Biosphere Reserve [16]–[20].

### Perimeter section between points No. 7 and No. 8: a critical mapping out

According to the Decree 2187 the ZITT boundary section between points No. 7 and No. 8 has to run exactly along the riverbed of the Rio Nashiño and consequently following the bottom valley line. On the contrary, the official cartographic representation of the ZITT boundary [17], [71], [80] seems to have mapped out a different limit line on the section close to point No. 7.

To validate this section of the limit by remote sensing technologies, a geospatial analysis has been developed using both DEM cartography (SRTM and GDEM maps) and satellite scenes (Landsat 5 TM) in order to deeply investigate the terrain morphology and the river dynamics in this area.

**Figure 4:**
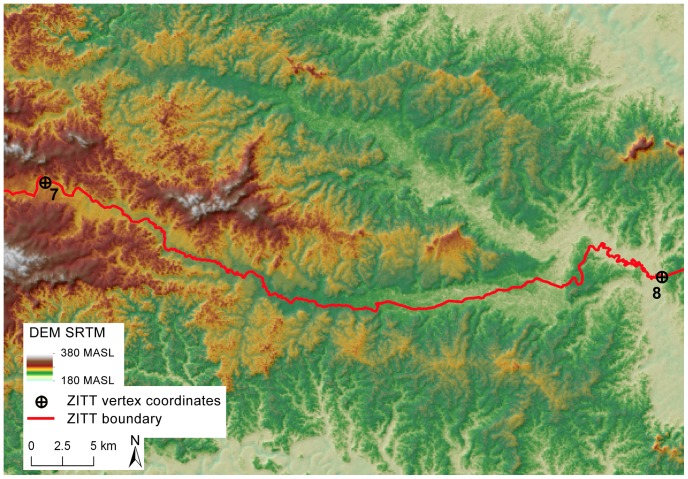
Digital Elevation Model (SRTM) analysis between points No. 7 and 8: a critical mapping out of the *Zona Intangible Tagaeri Taromenane* (ZITT). The DEM analysis of river dynamic clearly shows that the boundary section, rather than overlapping the Rio Nashiño river bed, drifts away from the river course running over a hills line upper slope. This mapping out violates both the terrain morphology and the same official text (Decree 2187, 2007).

To deploy a crossed spatial analysis we have examined four Landsat 5 TM satellites scenes in the years 1990, 2004, 2005, and 2008. The DEM analysis of river dynamics clearly shows that the boundary section drifts away from the Rio Nashiño riverbed running over a hill's ridge (Figures 4, S4).

**Figure 5:**
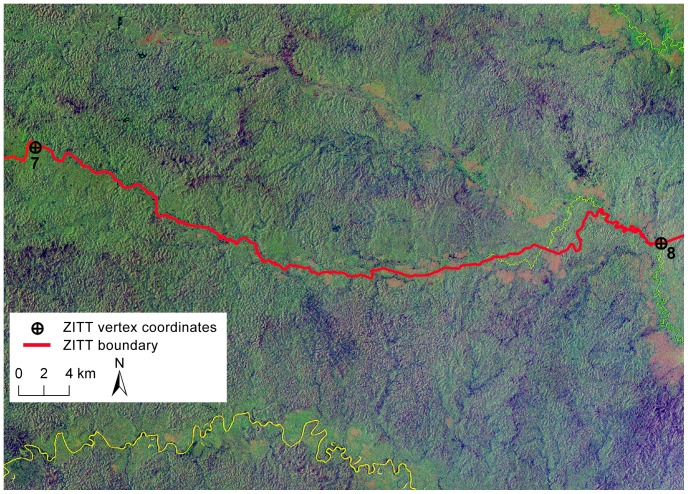
Spatial analysis of perimeter section between points No. 7 and No. 8 processing satellite scenes from Landsat TM 5. Landsat Thematic Mapper (TM) false color composite (4 blue, 5 green, 7 red) of the territory encompassed between points No. 7 and No. 8 in the southern sector of the Oil Block 31 (PetroAmazonas). Boundary section (red color line) does not match the Rio Nashiño river bed (yellow color line).

The Rio Nashiño river course is consistent with all the analyzed time series satellite scenes. Despite the previous cartographic base used to trace out the ZITT limits, this boundary section does not match with results obtained by remote sensing analysis of Landsat 5 TM satellite imagery (Figures 5, S5), SRTM DEM, nor Aster GDEM. Following its own trace violates both the terrain morphology and the same official text (Decree 2187).

### The Zona Intagible Tagaeri Taromenane and the Yasuní Biosphere Reserve: a synthesis map

The nine thematic variables assembled in the synthesis map give rise to the multiple territories setting up the Yasuní Biosphere Reserve space: the hydrocarbon dimension represented by concession, oil fields and wells for extraction and re-injection, protected areas recognized as IUCN category II for conservation of biodiversity by Yasuní National Park [65] and the Waorani indigenous territory by the titled-land (Waorani Ethnic Reserve), and finally the *Zona Intangible* for the Tagaeri Taromenane. Moreover, geodata about the historic incidents between Tagaeri Taromenane and external actors has been included in the map.

The hydrographic network (unfortunately the map presents a spatial gap in the south-east sector of the Yasuní Biosphere Reserve due to the unavailability of data) has been included in the map background because sections of river are often used as a “natural boundary” to delimitate protected areas (Yasuní National Park, Waorani Ethnic Reserve and ZITT) and they also play a crucial role in the indigenous territory building [56], [81]–[83]. Furthermore, according to anthropological studies, we considered Tagaeri Taromenane as indigenous groups who traditionally establish their territory along “inter-fluvial” spaces [3], [65], [84].

The road network is represented by the complex of the Via Auca (at the west sector, built in 1972), Via Maxus (north-west sector, built in 1994), Via “Oxy” (built in 2003 by the Occidental Petroleum company), and the Via “Petrobras” (built in 2004 by the homonymous Brazilian oil company). All the roads in the Yasuní Biosphere Reserve are important terrestrial infrastructures for hydrocarbon reserves exploitation [38], [85], [86].

**Figure 6:**
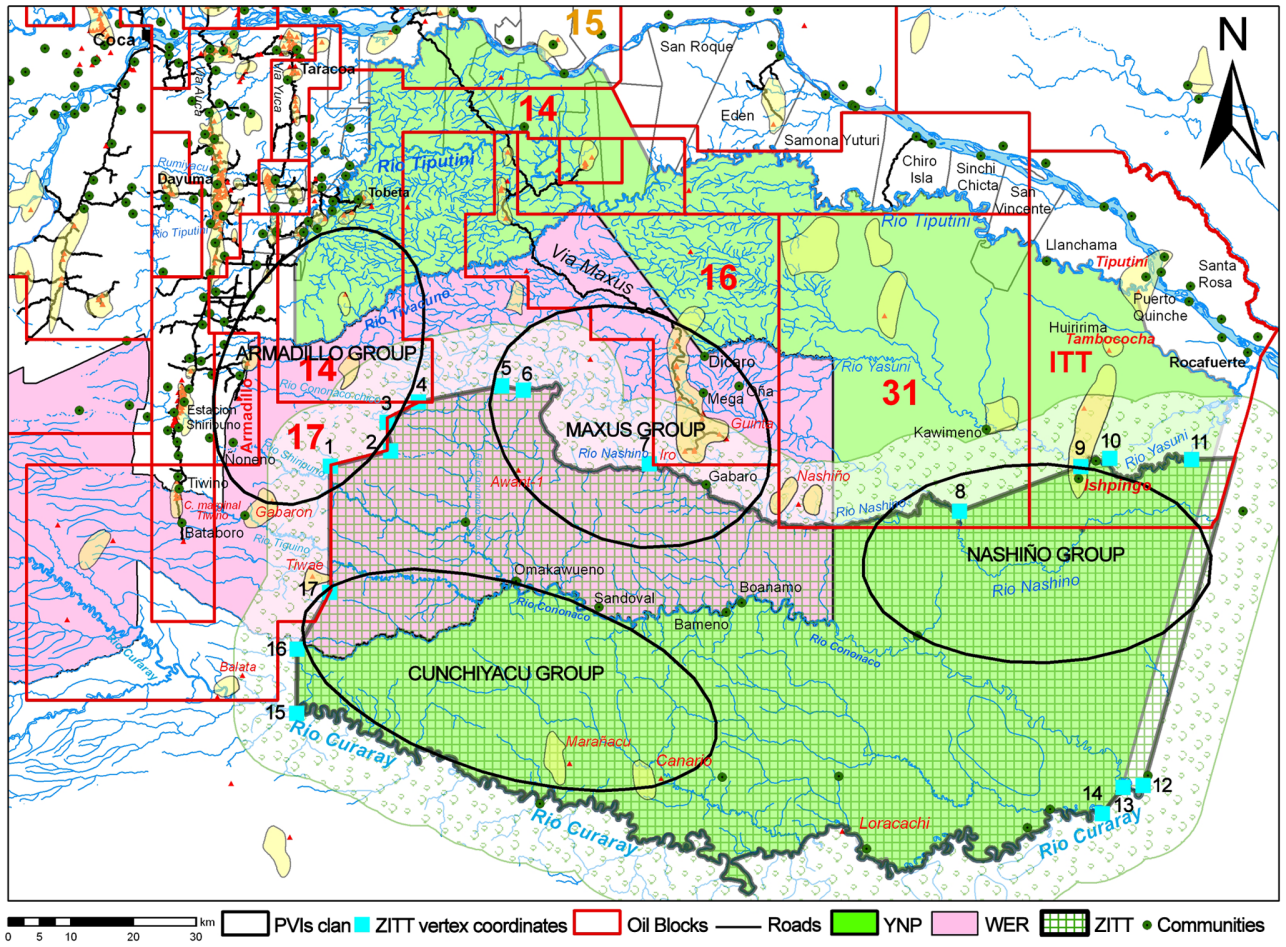
Synthesis map: Yasuní National Park (YNP), Waorani Ethnic Reserve (WER), *Zona Intangible Tagaeri Taromenane* (ZITT) and oil blocks. The overlapping systems of territorial complexity in and around the Yasuní Biosphere Reserve: the Zona Intangible (ZITT) is geographically adjoined with productive oil concessions area at the north-west side; on the contrary the north-east sector overlaps both with oil blocks No. 31 (operated by the Petroamazonas national company) and that ITT block (Ishipingo-Tiputini-Tambococha block, Secreteria de Hidrocarburos of Ecuador). The spatial distribution of the Tagaeri Taromenane clans is partially outside the ZITT perimeter in the cases of the Nashiño and the Maxus groups; the Armadillo group is completely outside the ZITT boundary overlapping oil blocks No. 14 and No. 17. Their home range overlaps several titled and untitled land of mestizo (colonos) farmers and non-autochthonous indigenous settlements in the Dayuma and Ines Arango districts, implicating a sensible proximity with roads system related to the Via Auca main axis. The presence of the Armadillo group in this area is also confirmed by recent violent contacts between the Hormiguero oil platform and the Armadillo oil field.

As it is shown in the synthesis map (Figure 6), presently the ZITT is geographically adjoined with productive oil concessions confirming the overlapping territorial policies. GIS analysis of spatial relationships between oil block areas and the *Zona Intangible* demonstrates that – except with blocks No. 16, No. 14 and No. 17 which seem to cut out the same ZITT perimeter – hydrocarbon concessions No. 31 (Petroamazonas) and the ITT block (managed by Secretaria de Hidrocarburos of Ecuador) result in, at the present time, an overlap of 8,542 and 9,952 ha (Figure 6; Table 1). It is relevant to clarify that the actual spatial dimension of oil blocks reflects the most recent changes operated by SHE in 2011 [87]. Before these relevant changes, previous GIS analysis had calculated a wider overlap of 84,789 ha by the overall oil blocks, corresponding to 10% of the ZITT. Areal overlap becomes greater if we consider the 10 km buffer zone of ZITT – 190,929 hectares, corresponding to 22,7% (Table 1) [20].

**Table 1:** Oil Blocks overlaps on the Zona Intangible Tagaeri Taromenane (ZITT) and the Buffer Zone (10 km).

Oil Blocks^a^	Total area Ha	ZITT Ha	Buffer Zone Ha	ZITT Buffer Zone Ha	ZITT %	Buffer Zone %	ZITT and Buffer Zone %
**ITT (Secretaria de Hidrocarburos de Ecuador)**	192,255	29,818	37,621	67,439	15,5	19,5	35
**31 (Petroamazonas)**	201,556	9,954	43,477	53,431	5	21,5	26,5
**17 (Petrooriental)**	186,186	0	50,720	50,720	0	27,2	27,2
**14 (Petrooriental)**	201,090	0	12,030	12,030	0	6	6
**16 (Repsol-YPF)**	130,642	0	13,715	13,715	0	10,5	10,5

Areal measures (hectares) and corresponding percentage. Data on oil blocks refers to the last updates of the 10^th^ oil concession licensing round.

GIS analysis of the spatial distribution of the four Tagaeri Taromenane clans [88] shows geographical weaknesses of the ZITT perimeter and the Buffer Zone in protecting the PVIs territory (Figure 6). As it is disclosed in the synthesis map, the ZITT area does not completely fit with the home range of Tagaeri Taromenane. The Cunchiyacu group is settled within the Curaray and Cononaco rivers within the ZITT. In the case of the Maxus and Nashino groups, the home range of these two clans is partially out of the ZITT area. On the contrary, the Tagaeri Taromenane Armadillo clan is definitely out of the same perimeter of the ZITT, completely overlapping oil blocks No. 14 and No. 17 (Figure 6) which are confirmed hydrocarbon reserve areas to be exploited by the Petrooriental oil company [89], [90].

Furthermore, the Armadillo clan home range overlaps several titled and untitled lands of *mestizo* (colonos) farmers and non-autochthonous indigenous settlements in the Dayuma and Ines Arango districts, implicating a sensible proximity with roads system (Figures S1, S2) related to the Via Auca main axis [38].

The fact is a crucial zone in which different actors and different territory projects are at stake is also confirmed by the dramatic contacts between *colonos* and uncontacted people in 2009. Only 400 meters from the Hormiguero extraction platform (oil blocks No.17), a Tagaeri Taromenane group speared to death three local farmers [3], [27], [28], [91].

The Nashiño and the Maxus groups are partially outside the ZITT perimeter overlapping three oil blocks (No. 14, No. 17, No. 16, No. 31 and the ITT). In the case of the Maxus group most of their territory, having a home range of roughly 30 km, is outside the ZITT within the oil blocks No. 16 and No. 14. Within block No. 16 the news documented a possible contact in March 5th (2013) (under verification by Ecuadorian Authorities) confirming it is a critical area [29], [92], [93]. This spatial distribution of these clans is also confirmed by previous anthropological analysis on the Tagaeri Taromenane issue [2], [3], [28], [84], [94], in which several witnesses by the Waorani communities, settled within the same oil blocks No. 16, report the presence of uncontacted people in this precise area. Moreover, qualitative field based research by Colleoni and Proaño refer to signs of presence by footprints and typical symbolic territory demarcations (i.e. crossed broken branches) in the area of the Rio Dicaro sources, between oil blocks No. 14 and No. 17., identifying a mobility space of uncontacted people that are used to cross from Rio Dicaro to Rio Yasuní (Figure 6). This behaviour is also confirmed by the database of Ministerio de Justicia y derechos Humanos related to the operation derived from direct survey and patrols of the ZITT and surrounding areas.

**Figure 7:**
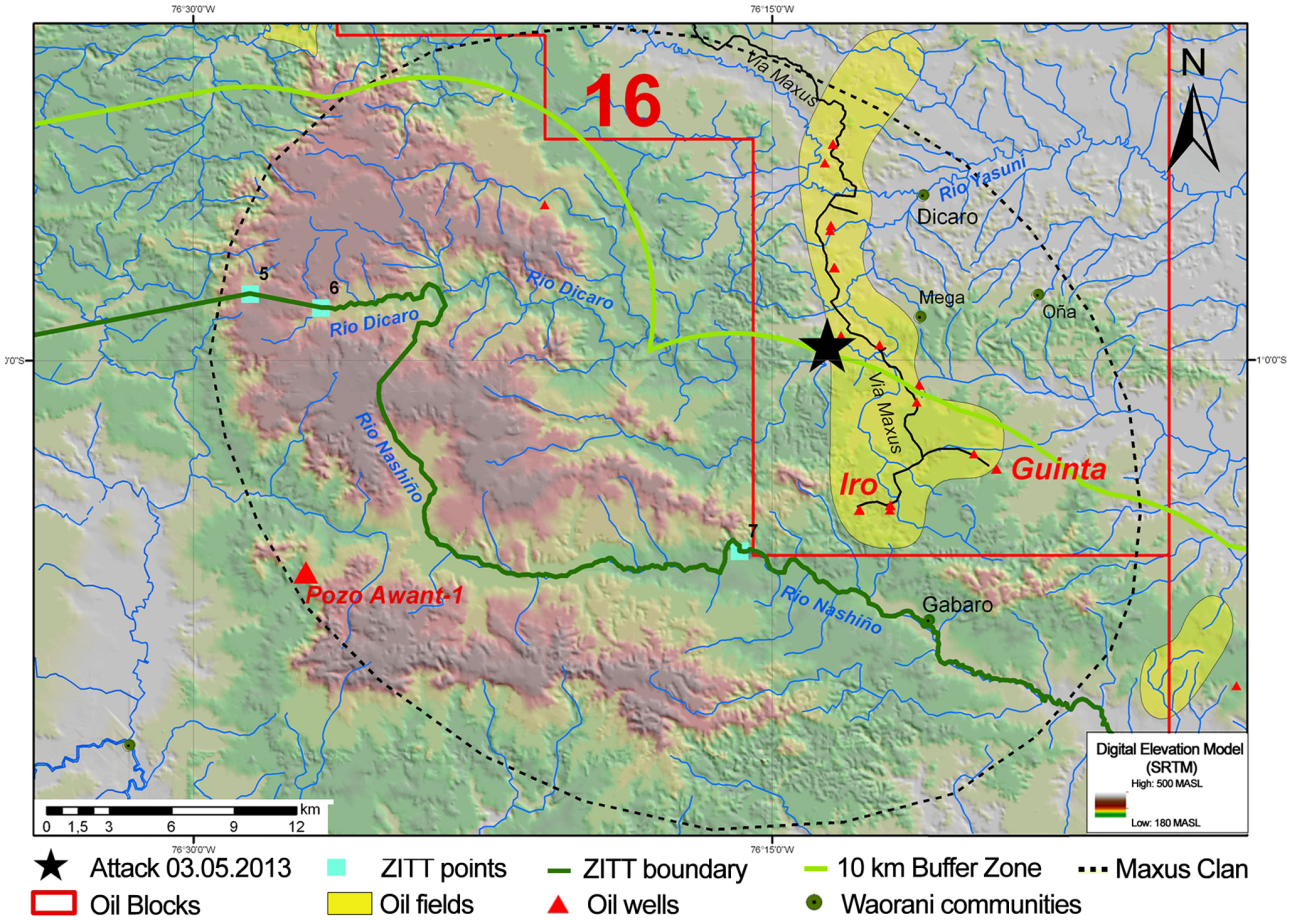
Synthesis map of the critical zone around points No. 5, 6 and 7 of the perimeter section: spatial relationships between oil production (block 16), home range of uncontacted Maxus group, and the spot of the Tagaeri Taromenane attack occurred on the 5^th^ March 2013. This synthesis map of the critical zone defined by points No. 5, 6, 7 of the *Zona Intangible* show sensible spatial relationships between oil production (blocks, fields and wells), the home range of uncontacted Maxus clan, the ZITT perimeter and the Waorani communities settlements. Spatial relationships between oil production areas and the *Zona Intangible* perimeter section are clearly shown in the map. The attack has major significance in terms of its location since it occurred near the geographic inconsistencies highlighted by this paper: about 100 meters from the Wipsi-1 oil platform, 10 km from point 7, almost 20 km from point 7, along the border of the Buffer Zone. Background raster map has been assembled using a SRTM DEM model to also show geomorphological characteristics of the area.

A synthesis map of the critical zone defined between points No. 5, 6, and 7 of the ZITT has been produced. Geospatial data on hydrocarbon concessions (10^th^ licensing round), oil fields and wells, Waorani communities, and the home range of Tagaeri Taromenane have been assembled using a SRTM DEM map to also show geomorphological characteristics. This map shows spatial relationships between oil production areas, the ZITT perimeter, and the Maxus uncontacted group (Figure 7).

### Drawing a new map: recognizing the spatial patterns of Tagaeri Taromenane

From the perspective of the geography discipline, the territory is not just a polygon closed by a perimeter linking the adjoining areas. Territories are based on junctions, corridors, multiple and non-continuous polygons, centres and peripheries, intensity and directions of functions, multiple uses of natural resources, and co-existence of actors [56], [81], [82], [95]–[97]. As it as shown by the geographical analysis of the ZITT, every geometrical space may include multiple territories, often conflicting each other.

**Figure 8:**
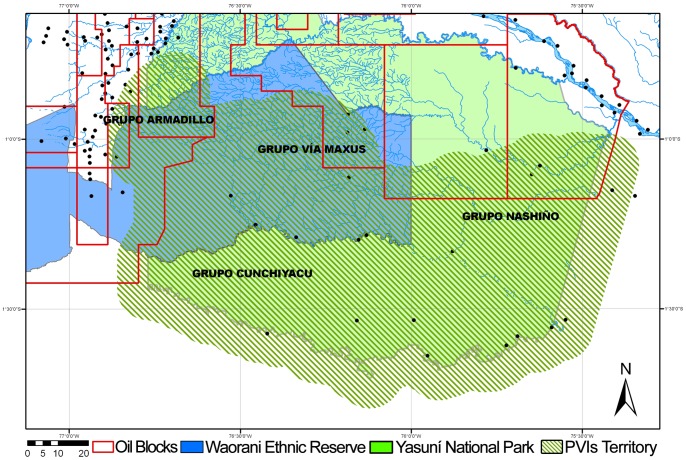
Synthesis map of the *Zona Intangible.* Recognizing the complex territory of uncontacted Tagaeri Taromenane: from geometry to geography. The map results from the combination of human right policies *(Zona Intangible Tagaeri Taromenane)* representing also the Buffer Zone (normally avoided in official maps), the oil concessions for hydrocarbon production and the spatial patterns of Tagaeri Taromenane (combining the results of data about paths and presences). Considering the power of images in shaping territorial representation, cartography science has the responsibility in formulating more complex discourses about controversial territories.

In a geographical perspective, territories are produced not only by physical intervention, but also (and before physical intervention) by images and particularly maps since they are the strongest way to produce imaginary geographies [98]–[102]. Normally maps presenting the ZITT are produced in the framework of the Westphalian territorial idea based on a rigid geometry of surface and perimeter which is the classical and most common representation of the ZITT. Figure 8 tries to produce a map representation closer to human right policies and the spatial patterns of Tagaeri Taromenane. First of all limits are abolished. Second, there is a representation of the buffer zone (normally not communicated). Third, a territorial continuity between the nuclei of the four groups and the official ZITT has been represented. This representation shows that the traditional geometric ZITT seems unable to contain the territory reconstructed from the traces of practical territorialization implemented by Tagaeri Taromenane. The decision to use a texture instead of a strong tick colour or an empty area with a marked perimeter is a typical choice from a wide array of tools available to cartographic science. Its usage stresses the communication message of a cultural geography of network relationships among societies and ecosystems [103]–[106].

This map is only a first attempt to highlight how the recognition of indigenous territory in the Amazon basin needs to implement geographical approaches in order to deal with the complex dynamics of territories and territorialities in relation to actors and conflicts.

## Materials and Methods

To develop geographical analysis of the ZITT limits we primarily examined the Decrees No. 552 (1999) and No. 2187 (2007). The first one officially declares the No-Go-Zone as “an Intangible Zone for conservation of the homeland of the Waorani groups known as Tagaeri Taromenane in which it is forbidden in perpetuity whatever sort of extractive activities” (see the Spanish original text in supporting materials) while the second one determines the boundary of the ZITT providing geospatial information and detailed geomorphological description of the area [14], [15]. In the second Decree (2007) the ZITT is cartographically defined by 17 pairs of metrical coordinates conjoined to each other by straight lines or sections of river courses (Figure 1A). It also declares the Geographical System adopted to mark off the ZITT: Provisional South America Datum 1956 (PSAD-1956), 18 South Zone. The total area declared is 758,051 ha.

In order to validate the perimeter of the ZITT, we analyzed topographic sheets by the Military Geographic Institute of Ecuador (Instituto Geografico Militar, IGM) at different scales (1:50,000, 1:250,000). The IGM cartography at a 1:50,000 scale consists of four sheets named as Rio Bahameno [107], Rio Yasuní Este [108], Rio Nashiño [109], and Rio Yamino [110]. The sheets adjoin each other to form four quadrants including, in their common corner, the perimeter between points No. 5, No. 6, and No. 7. The sheets are provided of geographical metadata defining as projected into a UTM 18 South, Datum WGS 1984 except the Rio Nashiño map that is projected in the Zone 17 South. All four sheets have been assembled using aerial photography mainly taken in the year 1986 while the “Yasuní” sheet is derived by photographic coverage from 1963 and the “Rio Bahameno” sheet from 1976. Contour lines show an equidistance mainly of 20 meters and occasionally of 10 meters. These values are obtained by subtracting a constant of 30 meters of altitude assuming an average canopy of the MTF of 30 meters high.

Altimetry points show a distribution average of 0.6 values per km^2^, calculated by three information aliquots of 66 km^2^. Geodetic and trigonometric points are rare most likely due difficulty of accessing to the Amazon territory.

To perform a comparative cartographic analysis we also used the 1:250,000 IGM sheets named Shushufindi [111] and Curaray [112]. Unfortunately, the Military Geographic Institute does not cover the eastern territory of the ZITT by the other two adjoining quadrants. However, the “Shushufindi” sheet represents a more detailed cartographic landscape compared to the ones at 1:50,000 scale. On the contrary, the Curaray sheet is definitely poor, without any basic metadata. These topographic sheets were produced in the year 1998 and are basically derived from the 1:50,000 scale sheets. Therefore, we do not expect different information according to the larger scale. In any case, both the 1:250,000 scale sheets represent a poor and incomplete hydrographic network. For instance, there are some river sources ending at the border of the sheet and not continuing onto the adjoining one.

In order to analyze the spatial relationships both of catchment's divides and hydrographic network related the ZITT perimeter we acquired raster thematic cartography (Table S2). Three types of Digital Elevation Models (DEM) maps have been used to deeply investigate both the terrain morphology and river dynamics [113]–[116]: an IGM-DEM map [117], a Shuttle Radar Topographic Mission (SRTM) map [79], and a GDEM derived by the Aster satellite [78].

The IGM-DEM map presents a geometric resolution of 30 meters (one spot elevation every 900 m^2^), the SRTM map of 92 meters (one spot elevation every 8,400 m^2^), and the GDM Aster satellite of 30 meters. The SRTM elevation spot derives directly by radar interferometry technique. On the contrary, the IGM-DEM map is obtained not by remote sensing technology, but by cartographic data included in maps at 1:50,000 and 1:250,000 scale.

The remote sensing data from the Aster satellite (GDEM) presents a higher geometric resolution to the ground – 30 instead of 90 meters – but they show several false records due to the intense land cover of the MTF. Therefore, these satellite derived maps present a repetitive error more extensive in the representation, but homogeneous and, consequently, tolerable.

Moreover, Landsat satellite imagery has been used to validate some uncertain segments of the perimeter represented by river course sections. Three satellite time-series of Landsat Thematic Mapper (TM) false colour composite (4 blue, 5 green, 7 red) [118] of the territory encompassed between points No. 7 and No. 8 in the southern sector of the Oil Block 31 (PetroAmazonas) have been acquired. The Landsat satellite scenes acquired are from the years 1991 and 2005 (2^nd^ of July and 28^th^ of August respectively). For a complete overview of the raster cartography used in the spatial analysis see Table S1.

Other geodata has been used to perform the spatial analysis of the ZITT perimeter together with other key components shaping the Yasuní territory in order to have a systemic understanding of the complex territorial processes taking place in the area [20], [96].

Geospatial information on protected areas (Yasuní National Park, Waorani Ethnic Reserve), hydrographic network, and urban centres are from institutional web sources and were publicly available at the time of the submission. These include the World Database on Protected Areas (UNEP-WCMC, IUCN World Commissions on Protected Areas, 2012) [119] and the EcuadorIan IGM Geoportal. The official ZITT perimeter and geographical data on Tagaeri Taromenane clans distribution have been acquired from the Ministerio de Justicia, Derechos Humanos y Cultos of Ecuador (2012), who from October 2010 holds the Plan of Precautionary Measures for the Protection of Isolated Indigenous Peoples of Ecuador (Plan de Medidas Cautelares, PMC) [88].

Road network of Ecuadorian Amazon was taken by the Instituto Nacional para el Eco-Desarrollo de la Region Amazonica Ecuatoriana (ECORAE) in 2008 [120].

Oil fields and oil wells have been acquired by the ECORAE institution (2008). Oil blocks with the latest changes (2011) of the 10th hydrocarbon licensing round have been derived by digitizing oil maps released in 2012 by the *Secreteria de Hidrocarburos Ecuatoriano*s (SHE) [87]. An overview of characteristics of all vector geodata used in the spatial analysis are shown in Table S3.

The main tools to conduct cartographic and spatial analysis of the ZITT have been Geographical Information Systems (GIS) and remote sensing technologies. GIScience approach has been used to collect and elaborate different types of data, as long as spatially explicit, referring both to the biophysical components and to the anthropic one, so that cartographic outputs and synthesis maps make visible spatial relationships between relevant components of environmental systems and that of the anthropic one [121]–[124].

All the anthropic, physical, and geographical data collected have been integrated in a GIS database in order to manage spatially explicit, temporally referenced, and thematically differentiated information.

Spatial analyses are based both on a critical examination and integration of the physical and thematic cartography previously acquired and on GIS elaboration in order to produce analytical and synthesis maps showing the following data: hydrographic network, river basins, protected areas (Yasuní National Park, Waorani Ethnic Reserve, ZITT) and buffer zones, urban centres and local communities, road network, and the spatial dimension of the hydrocarbon exploitation (blocks, oil fields and wells). Moreover, we have also acquired spatial anthropological data concerning the Tagaeri Taromenane as the family clans spatial distribution [88].

ArcGIS^TM^ 10.1 software has been used for GIS analysis mainly conducted by spatial functions such as overlay, geometrical interception, linear, and area measurements. Raster data analysis has been carried out using IDRISI-GIS software.

To validate important data such as spatial information of key rivers, local communities and roads the GPS ground-truth methodology has been adopted during fieldwork activities (2005, 2006, 2010, 2011) [38] [125].

## Conclusions

Results of GIS and remote sensing analysis, fieldwork data, and examination of all the available sources show different critical issues in the Decree 2187 when it comes to defining the perimeter of the ZITT. They belong both at an interpretation level and, therefore, in the same cartographic representations. On the other side, the policies implemented in the ZITT and in the Buffer Zone show conflicting territorial objectives.

During the revision of this article, in the night between the 4^th^ and 5^th^ of March 2013, the old Waorani man Ompore and his wife Buganey were speared to death in proximity of Iro oil field, in Block 16, which is operated by Repsol-YPF (Figure 6, 7). The attack should be confirmed, and at the moment investigation into the incident has been initiated by police and Ecuadorian authorities to ascertain the responsibility of Tagaeri Taromenane groups. Information collected by Waorani relatives of Ompore Omeway and Buganey Cayga refer to repetitive requests by Tagaeri Taromenane directed to Ompore to act with the oil company to stop oil extraction, pollution of water and air, and noise [92], [93], [126]. Meanwhile, a group of Waorani from the Yarentaro community organized an expedition to avenge their dead relatives, entered the ZITT, and killed approximately dozens of Tagaeri Taromenane as well as abducted two uncontacted youths. This fact, confirmed by an official twitter account of Ministerio de Justicia on the 3^rd^ of April 2013, is also under investigation.

These two dramatic events should be deeply analyzed in order to understand the complex relationship among uncontacted Waorani groups and the wide group of Waorani, the discontinued presence of the State, the role of Waorani organizations, and the deterministic approach of some NGO advisors.

The incident of 5^th^ of March also demonstrates the long and complex process to enact human right policies in a controversial and complex territory. Moreover, the attack has major significance in terms of its location since it occurred near the geographic inconsistencies highlighted by this paper: about 100 meters from the Wipsi-1 oil platform, 10 km from point 7, almost 20 km from point 7, along the border of the Buffer Zone (Figure 7).

So it is interesting to analyze how the limit line of the ZITT runs when it moves – close to point No. 6, from west to east direction – within this zone inward the oil block 16 (Repsol-YPF): after a sudden curve upper slope it drastically runs to south leaving the oil blocks by the Nashiño river, setting up a kind of cartographic enclave by a “bird beak” shape (Figure 7). After considering previous anthropological studies on Tagaeri and Taromenane in this area, one can logically ask the question: is the ZITT boundary line between the Dicaro and Nashiño rivers shaping an ethnical enclave as well?

Using a systemic approach to better define this issue, it is relevant to also take into account the geographical position and the same history of the ZITT (Figure 6).

During three years of the delimitation process (2004–2007), the Technical Commissions established by the Environment Ministry of Ecuador had to deal with blocks for oil exploitation which extensively overlap the northwest sector of the ZITT (Figure 6). This is the case of oil block No. 14 and No. 17 (Andes-Petroleum Company, China) which presented an overlap of 2,719 and 24,534 ha, respectively, in the same area of the ZITT, namely 1.4% and the 49.6% of each oil concession (Table 1) [64].

Due to a possible and relevant reduction of oil block areas during the ZITT delimitation process, the Andes-Petroleum company suggested to the Ministry of Energy (Ministerio de Energia y Minas) and its Environmental Department (DINAPA) to modify the perimeter in order to facilitate oil exploitation in the area maintaining direct access to the proved oil field called Awant-1. Previous GIS analysis [20] showed the “cartographic suggestion” of Andes Petroleum to create – according to its productive requirements – a special corridor to exploit the Awant-1 oil field (Fig. S6) which is localized 12,8 km to south point No. 6, inside the same ZITT area (Figure 7).

All of these geographical elements display the complexity of the Yasuní Biosphere Reserve in which different projects of territory – and, therefore, different modalities to use natural resources – are overlapped and often in conflict each other. A geography of energetic resources marked in maps by productive zoning (oil blocks) is superposed to that of protected areas (Yasuní National Park), to that of farmers settlements (Agrarian Reform), to that of indigenous territory (Waorani Ethnic Reserve), and finally to that of the ZITT [38], [51], [56].

The same delimitation of the ZITT induced a national and international debate on uncontacted people and their territorial rights. Aguirre (2007) stresses that since first steps of delimitation (2004) the process has been sinuous and thorny, made by consultation and negotiations, *in primis* with oil companies operating in the area of the ZITT. Later with officers of United Nation and IUCN, with the Waorani indigenous Organization (NAWE) and the National Indigenous Confederation of Ecuador (CONAIE), and finally national and international NGOs, without avoiding the weak but existing pressures from the local *colonos* (settled farmers of agrarian reform).

In general, beyond the technical issue to map out a perimeter for the ZITT, anthropological studies argue that a lack of understanding of territory and the same territoriality of the Tagaeri Taromenane is one of the main factors of the weakness in delimitating an *Zona Intangible* [2], [3], [6], [25], [41].

It is not just a technical issue considering the geographical and the biophysical characteristics of an area placed in the core of the Amazon region. It is a remote zone in which the Moist Tropical Forest as well as the dense and homogeneous hydrographic network, both set up a territory that is difficult to study, understand, and control. Therefore, mapping out the boundaries by “remote” analyses, using unverified geospatial data and not considering the complex arena in which territorial dynamics and the same territoriality of the Tagaeri Taromenane take place, is very difficult without holistic approaches and knowledge based on fieldwork.

Moreover, if we consider that the Decree 2187 uses “natural boundaries”, such as section of rivers, to delimit the ZITT perimeter, cartographic operations become more complicated, especially if the geospatial data is not precise, they present data gaps, or relevant geographic errors.

On the other side, a ZITT perimeter only based on straight lines could be easily traced out. However, in tropical forest territory, whatever border marked out that does not have clear natural spatial references, such as rivers or hills, results in a concealed boundary. A ZITT cartographically perfect will be produced, but at the same time it is invisible on the ground to both residents and to the people “from outside”.

A scientific contribution in the ZITT debate is necessary to make visible this territory (physically and graphically) to different components of Ecuadorian society because the ultimate objective of this policy is the right to liveable place of Tagaeri Taromenane uncontacted people.

In a structural context where media debate about the Amazon as “space of exception” [100], and ZITT territoriality is disputed, it is important to remember what “*The protection guidelines for indigenous peoples in isolation*” issued in May 2012 by the Office of the High Commissioner for Human Rights (OHCHR, 2012) states: “*While there is no consensus on the term to be used to refer to these people, internationally the most widely used is the concept of "isolated peoples”, in some countries they are known as, inter alia, free people, uncontacted, hidden, invisible, in voluntary isolation. Despite the different formulations, all refer to the same concept “isolation was not a voluntary option but a survival strategy (par. 9)”.*

## Supporting Information

Figure S1 **Land cover map (Landsat ETM+, 2002) of the Via Auca territory and anthropic pressures on the** ***Zona Intangible*** **and the uncontacted indigenous group.** Deforestation processes developed by road systems around the Via Auca main axis, African Palm cultivations (yellow areas at the north sector), *Zona Intangible Tagaeri Taromenane,* Armadillo uncontacted clan and oil blocks.
(TIF)

Figure S2 **Expansion of the Via Auca road network towards the Yasuní National Park and the Waorani Ethnic Reserve.** Oil blocks (yellow lines), historic incidents (red stars) between uncontacted Tagaeri Taromenane groups and external actors, and protected areas. Road network in 2009 by black lines (MAE, 2009) and spatial evolution to the east in 2011 developed by GPS survey (red lines). The map also show the uncontacted Armadillo home range overlapping several oil blocks and the mestizo farmer settlements.
(TIF)

Figure S3 **Comparative GIS analysis of catchment's divides by different Digital Elevation Models.** Catchment's divides are obtained by three different DEM maps (IGM Ecuador, SRTM, GDEM). Even if they present some spatial differences, all of them confirm that points No. 5 and No. 6 belong to different river basins. The first one belong to the Rio Yasuni, the second one to the Rio Curaray.
(TIF)

Figure S4 **Details of the boundary section nearby point No. 8 which drifts away from the Rio Nashiño river bed.**
(TIF)

Figure S5 **Detail of the Landsat TM 5 satellite scene and the boundary section close to point No. 8.** The boundary section drifts away from the Rio Nashiño riverbed runs over a hill's ridge in two spots, violating both the terrain morphology and the same official text (Decree 2187, 2007).
(TIF)

Figure S6 **Andes-Petroleum company and the cartographic suggestion to modify the perimeter of the** ***Zona Intangible***. Andes-Petroleum company (China) suggested to the Ministry of Energy (Ministerio de Energia y Minas) and its Environmental Department (DINAPA) to modify the perimeter in order to facilitate oil exploitation in the area maintaining direct access to the proved oil field called Awant-1. This map shows the “cartographic suggestion” of Andes Petroleum to create –according to its productive requirements – a special corridor to exploit the Awant-1 oil field which is localized 12,8 km to south point No. 6, inside the same ZITT area.
(TIF)

Document S1 **Presidential Decree 2187 signed by President Alfredo Palacio in 2007.** This is the official document which establishes and maps the *Zona Intangible Tagaeri Taromenane* by given vertex coordinates and geomorphological description of the area.
(PDF)

Table S1 **Metric coordinates of the 17 given points by the Presidential Decree 2187 (2007) defining the Intangible Zone Tagaeri Taromenane.**
(PDF)

Table S2 **Geospatial raster data used in GIS analyses: coverage, typology, resolution and sources.**
(PDF)

Table S3 **Characteristics and sources vector geodata.**
(PDF)
