# Supplementary figures and images for: Uncontacted Waorani in the Yasuní Biosphere Reserve: Geographical Validation of the Zona Intangible Tagaeri Taromenane (ZITT)
Source: PLoS One. 2013 Jun 19;8(6):e66293. doi: 10.1371/journal.pone.0066293 (PMC3686793; doi:10.1371/journal.pone.0066293)

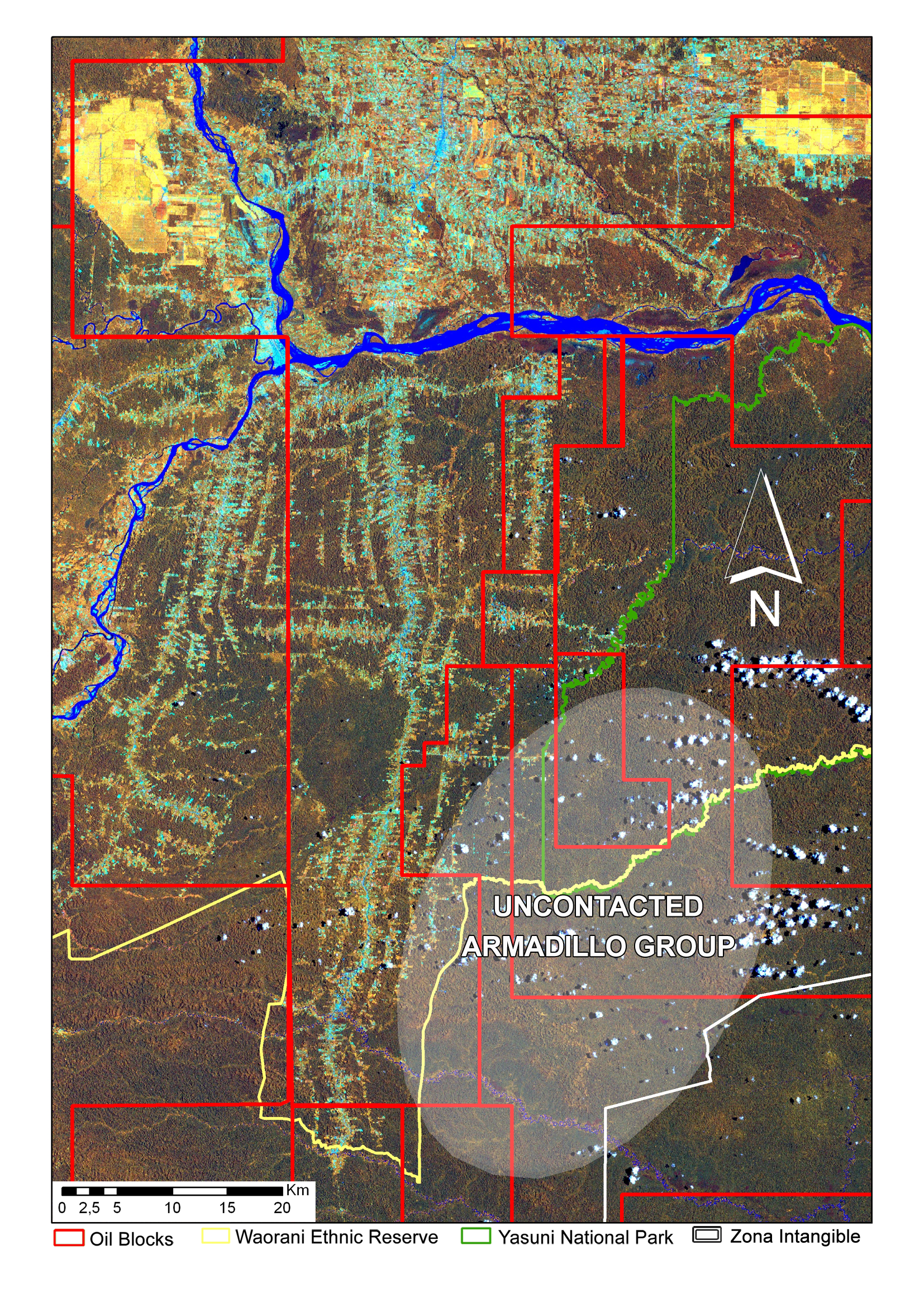

Supplement: Figure S1 — Land cover map (Landsat ETM+, 2002) of the Via Auca territory and anthropic pressures on the Zona Intangible and the uncontacted indigenous group. Deforestation processes developed by road systems around the Via Auca main axis, African Palm cultivations (yellow areas at the north sector), Zona Intangible Tagaeri Taromenane, Armadillo uncontacted clan and oil blocks. (TIF) [file pone.0066293.s001.tif]

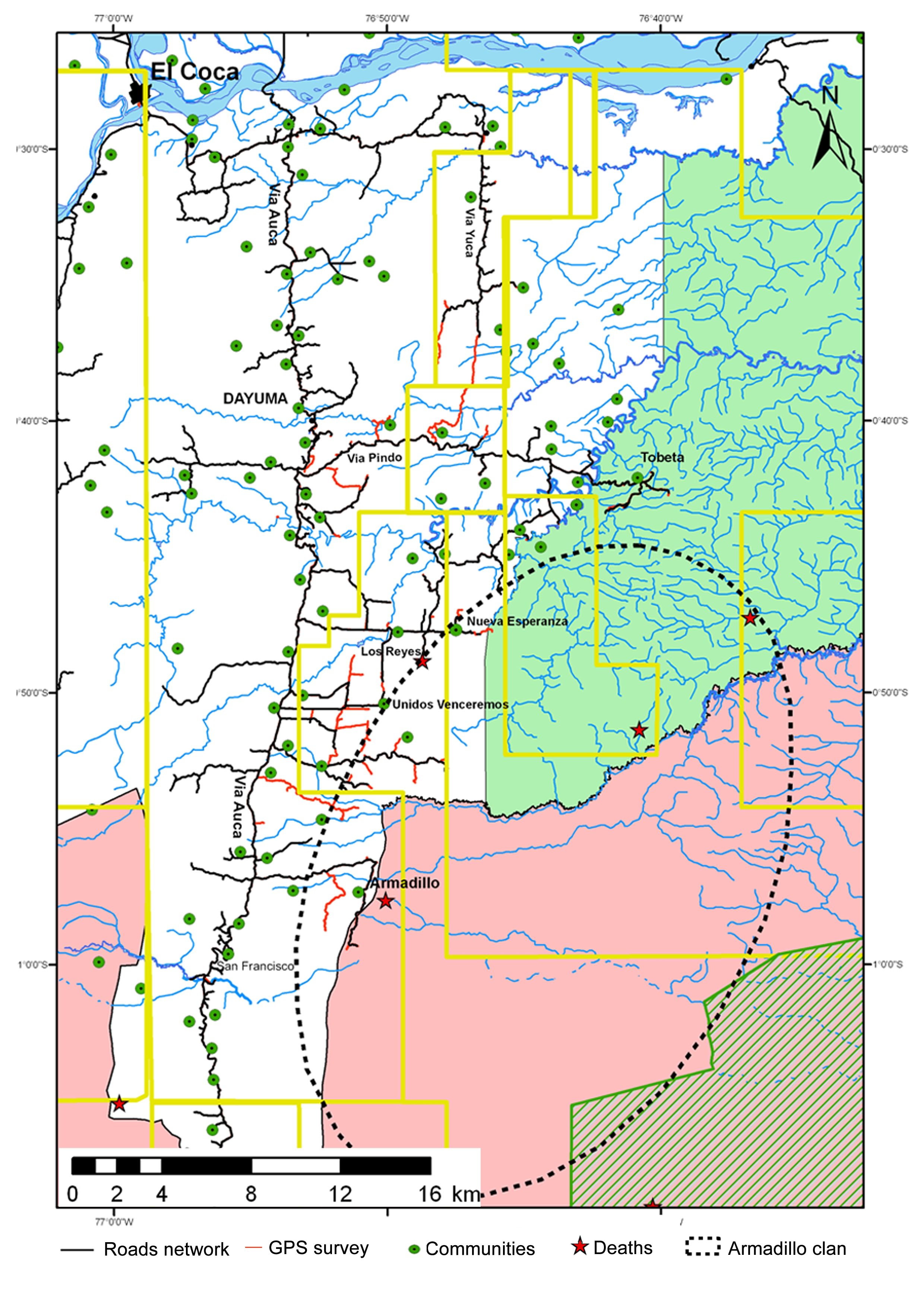

Supplement: Figure S2 — Expansion of the Via Auca road network towards the Yasuní National Park and the Waorani Ethnic Reserve. Oil blocks (yellow lines), historic incidents (red stars) between uncontacted Tagaeri Taromenane groups and external actors, and protected areas. Road network in 2009 by black lines (MAE, 2009) and spatial evolution to the east in 2011 developed by GPS survey (red lines). The map also show the uncontacted Armadillo home range overlapping several oil blocks and the mestizo farmer settlements. (TIF) [file pone.0066293.s002.tif]

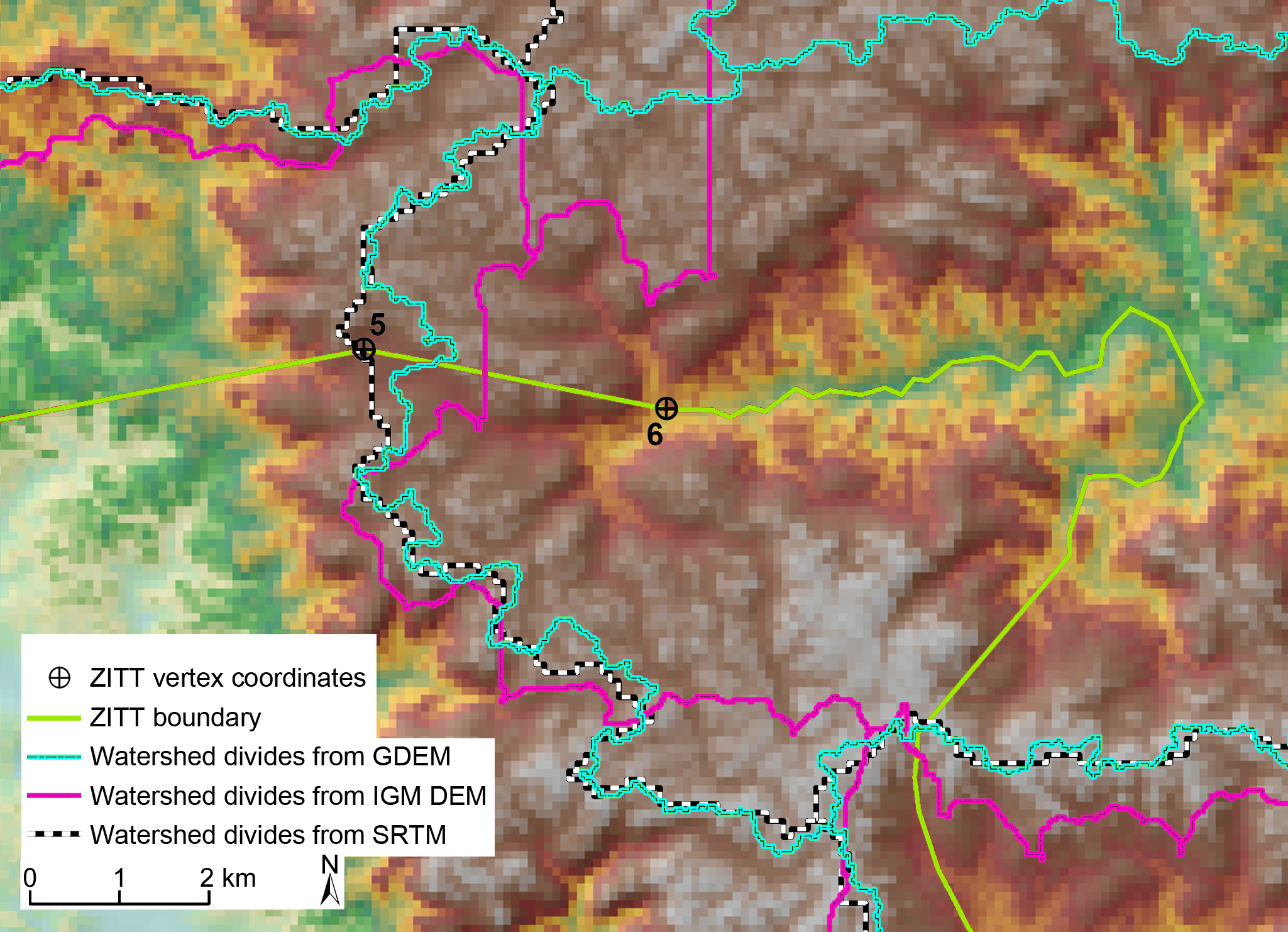

Supplement: Figure S3 — Comparative GIS analysis of catchment's divides by different Digital Elevation Models. Catchment's divides are obtained by three different DEM maps (IGM Ecuador, SRTM, GDEM). Even if they present some spatial differences, all of them confirm that points No. 5 and No. 6 belong to different river basins. The first one belong to the Rio Yasuni, the second one to the Rio Curaray. (TIF) [file pone.0066293.s003.tif]

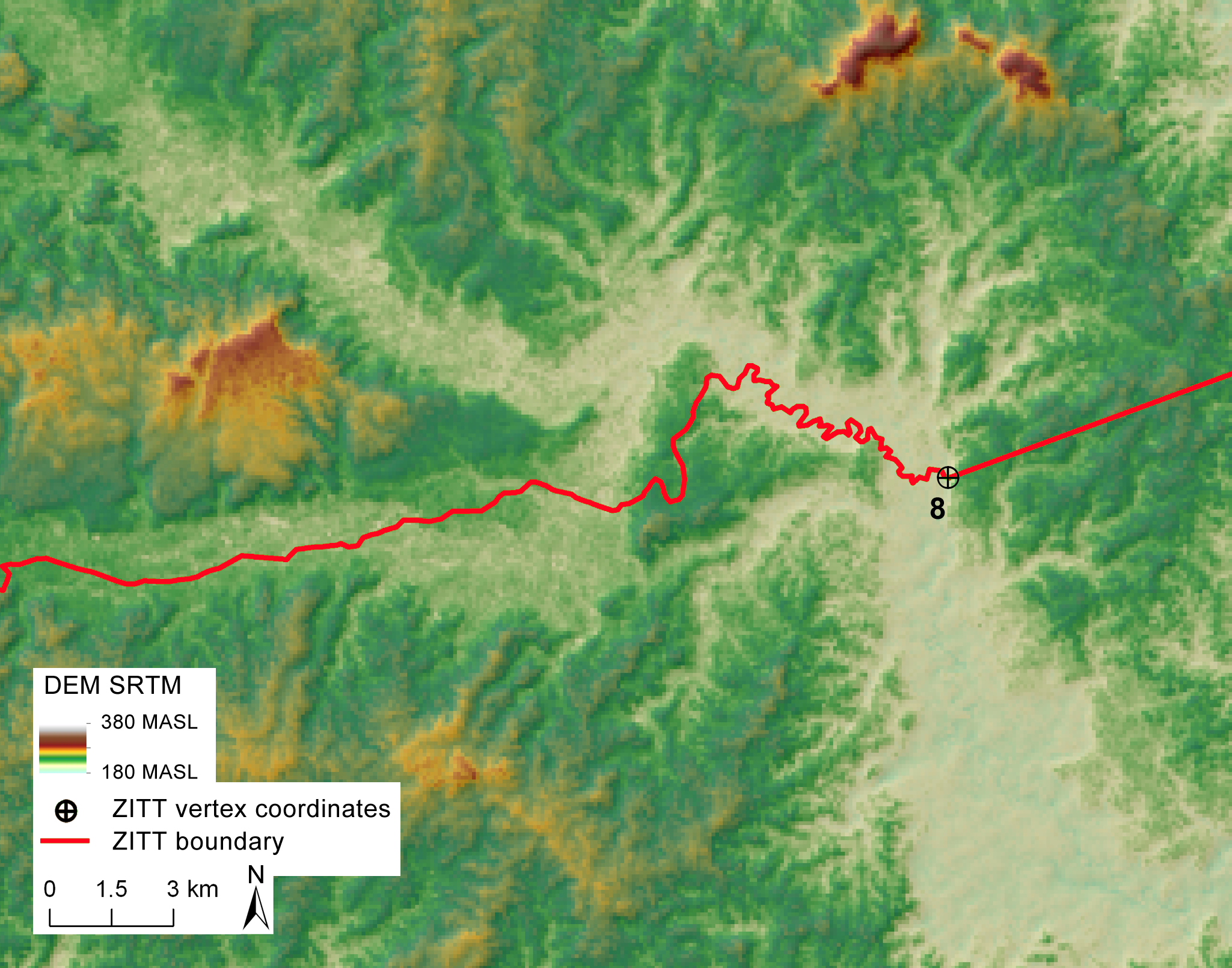

Supplement: Figure S4 — Details of the boundary section nearby point No. 8 which drifts away from the Rio Nashiño river bed. (TIF) [file pone.0066293.s004.tif]

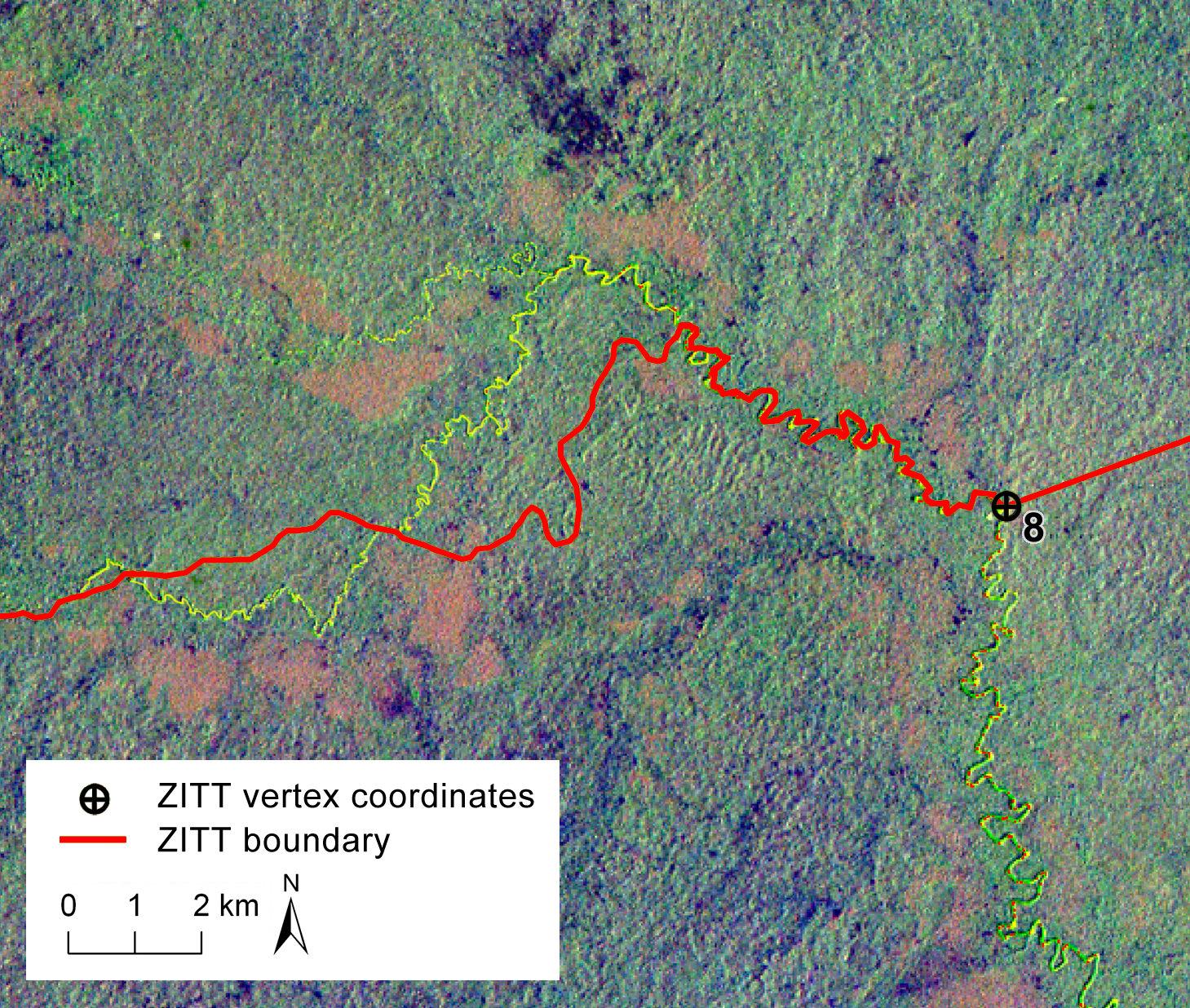

Supplement: Figure S5 — Detail of the Landsat TM 5 satellite scene and the boundary section close to point No. 8. The boundary section drifts away from the Rio Nashiño riverbed runs over a hill's ridge in two spots, violating both the terrain morphology and the same official text (Decree 2187, 2007). (TIF) [file pone.0066293.s005.tif]

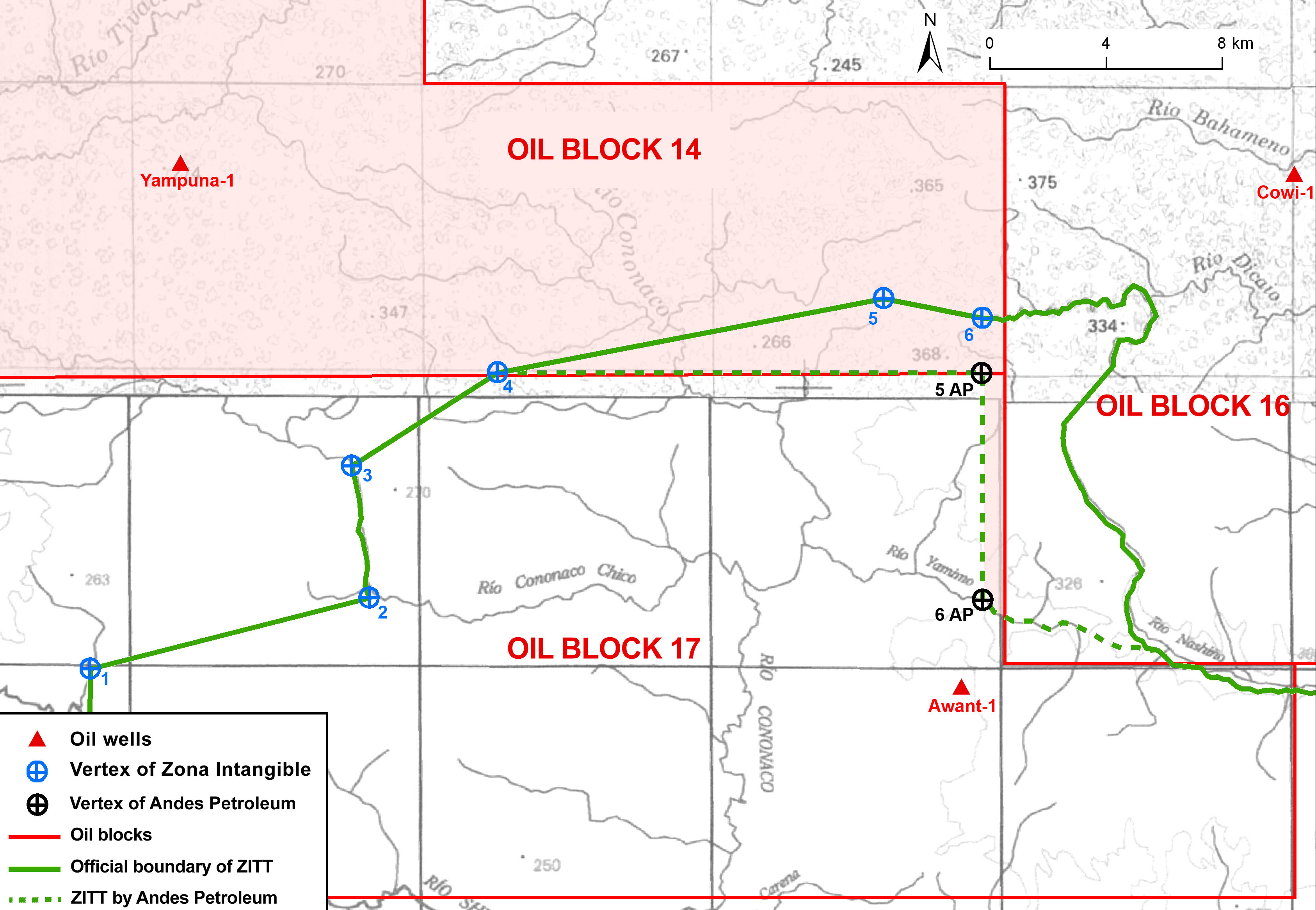

Supplement: Figure S6 — Andes-Petroleum company and the cartographic suggestion to modify the perimeter of the Zona Intangible. Andes-Petroleum company (China) suggested to the Ministry of Energy (Ministerio de Energia y Minas) and its Environmental Department (DINAPA) to modify the perimeter in order to facilitate oil exploitation in the area maintaining direct access to the proved oil field called Awant-1. This map shows the “cartographic suggestion” of Andes Petroleum to create –according to its productive requirements – a special corridor to exploit the Awant-1 oil field which is localized 12,8 km to south point No. 6, inside the same ZITT area. (TIF) [file pone.0066293.s006.tif]
