# Supplementary material for: Uncontacted Waorani in the Yasuní Biosphere Reserve: Geographical Validation of the Zona Intangible Tagaeri Taromenane (ZITT)
Source: PLoS One. 2013 Jun 19;8(6):e66293. doi: 10.1371/journal.pone.0066293 (PMC3686793; doi:10.1371/journal.pone.0066293)
Supplement: Document S1 — Presidential Decree 2187 signed by President Alfredo Palacio in 2007. This is the official document which establishes and maps the Zona Intangible Tagaeri Taromenane by given vertex coordinates and geomorphological description of the area. (PDF) [file pone.0066293.s007.pdf]

PRESIDENCIA DE LA REPÚBLICA DEL ECUADOR
SECRETARÍA GENERAL DE LA ADMINISTRACIÓN PÚBLICA
OFICINA DE DECRETOS Y TRAMITES ADMINISTRATIVOS

PARA: DIRECTOR DEL REGISTRO OFICIAL

CLASE: DECRETO

Nº 2187 **FECHA:** 3 de enero del 2007

ASUNTO: SE ENVIA POR DISPOSICIÓN DEL SR. SECRETARIO GENERAL EL DECRETO EJECUTIVO Nº 2187, PARA QUE SEA PUBLICADO EN EL REGISTRO OFICIAL, CON EL CARACTER DE URGENTE.

R E C E P C I O N

Recibido por:

Nombres y Apellidos | Firma

Fecha | Hora | Sello

566219.

N° 2187

ALFREDO PALACIO GONZÁLEZ

PRESIDENTE CONSTITUCIONAL DE LA REPÚBLICA

CONSIDERANDO

Que la Constitución Política de la República en su artículo 1 dispone que el Ecuador es un Estado social de derecho, pluricultural y multiétnico, así como también garantiza el derecho colectivo de los pueblos indígenas a no ser desplazados de sus tierras conforme lo establecido en el número 8 de su artículo 84;

Que es un derecho constitucional y un deber del Estado la protección de las culturas ancestrales selváticas de la Amazonía, y dentro de éstas, los grupos que se mantienen sin contacto con la sociedad, integrados según los conocimientos actuales, por los que han sido denominados Tagaeri y Taromenane;

Que existen diversas amenazas externas a causa de políticas de desarrollo (proyectos y megaproyectos hidrocarburíferos, mineros, viales, hidroeléctricos, forestales, agropecuarios, de recursos hídricos, privatización de los recursos naturales - aguas, bosques, biodiversidad), actividades ilícitas (extracción forestal, narcotráfico, minería, extracción de fauna y flora), la deforestación, la colonización así como por la presencia de agentes externos (organizaciones religiosas, turísticas, científicas, empresas de cine/televisión, aventureros, otros);

Que el convenio No. 169, sobre Pueblos Indígenas y Tribales en Países Independientes de la Organización Internacional del Trabajo, ha sido ratificado por todos los Estados que cuentan con pueblos indígenas aislados (Bolivia, Brasil, Colombia, Ecuador, Paraguay y Perú); y, por tanto, es de obligatorio cumplimiento como ley nacional en cada uno de estos países;

Que el Estado ecuatoriano es signatario de la Declaración Universal de los Derechos Humanos, el Convenio sobre Prevención y Sanción del Genocidio, la Declaración Universal sobre Diversidad Cultural de la UNESCO, la Convención de París sobre la Protección del Patrimonio Intangible y el Convenio de Diversidad Biológica;

Que es obligación del Estado el reconocimiento legal de los territorios originarios y tradicionales, y la condición de inalienabilidad, inviolabilidad, indivisibilidad, imprescriptibilidad de éstos, a fin de garantizar la integridad y continuidad física y cultural de los pueblos ocultos no contactados; para lo cual, el Estado debe garantizar la supervivencia física y cultural de los pueblos ocultos no contactados - Taromenane y Tagaeri, respetando la voluntad de grupos que desean permanecer aislados y desenvolverse conforme a sus propios valores y tradiciones;

N° 2187

ALFREDO PALACIO GONZÁLEZ

PRESIDENTE CONSTITUCIONAL DE LA REPÚBLICA

Que mediante Decreto Ejecutivo No. 552 publicado en el Suplemento del Registro Oficial No. 121 de 2 de febrero de 1999, se declaró como zona intangible de conservación vedada a perpetuidad a todo tipo de actividad extractiva, las tierras de habitación y desarrollo de los grupos Huaorani conocidos como Tagaeri, Taromenane y otros eventuales que permanecen sin contacto, ubicadas hacia el sur de las tierras adjudicadas a la nacionalidad Huaorani en 1990 y del Parque Nacional Yasuní; zona, que alcanza aproximadamente 700.000 hectáreas;

Que mediante Acuerdo Interministerial No. 092 suscrito entre el Ministro del Ambiente y el Ministro de Energía y Minas, publicado en el Registro Oficial No. 440 del 12 de octubre del 2004, se constituyó la Comisión para la delimitación de la zona intangible declarada mediante Decreto Ejecutivo No. 552 antes citado;

En ejercicio de las atribuciones que le confiere el numeral 3 del artículo 171 y el numeral 2 del artículo 179 de la Constitución Política de la República y la letra f) del artículo 11 del Estatuto del Régimen Jurídico Administrativo de la Función Ejecutiva,

DECRETA:

Art. 1.- Delimítase la zona intangible establecida mediante Decreto Ejecutivo No. 552, publicado en el Suplemento del Registro Oficial No. 121 de 2 de febrero de 1999, misma que alcanza 758.051 hectáreas (setecientas cincuenta y ocho mil cincuenta y un hectáreas), que se ubican en las parroquias de Cononaco y Nuevo Rocafuerte, cantón Aguarico, provincia de Orellana; en la parroquia de Curaray, cantón Pastaza, provincia de Pastaza, en los siguientes límites:

Inicia en el punto No.1 de coordenadas UTM 9880258 norte y 308703 este, sigue en dirección noreste una línea recta de 10.000 metros hasta alcanzar el Río Cononaco Chico en el punto No. 2 de coordenadas 9882711 norte y 318314 este, en donde toma una dirección norte, siguiendo el curso del mismo río hasta el punto No. 3 de coordenadas 9887248 norte, y 317709 este. Desde el punto No. 3 se dirige una línea recta de 6.140 metros en dirección noreste hasta el punto No. 4 de coordenadas 9890474 norte y 322749 este. Con la misma dirección continúa una línea recta de 13.500 metros hasta el punto No. 5 de coordenadas 9893028 norte y 336056 este; luego, sigue una línea recta de 3.500 metros en dirección sureste hasta alcanzar la unión de los tributarios del Río Bahameno (Baameno) en el punto No. 6 de coordenadas 9892355 norte y 339450 este.

N° 2187

ALFREDO PALACIO GONZÁLEZ

PRESIDENTE CONSTITUCIONAL DE LA REPÚBLICA

Desde el punto No. 6 sigue en dirección este por el curso del Río (Diicaro); luego, sigue aguas abajo por el Río Nashiño hasta el punto No. 7 de coordenadas 9880661 norte y 359579 este, desde donde continúa aguas abajo por el mismo Río Nashiño hasta el punto No. 8 de coordenadas 9873067 norte y 409111 este, a continuación sigue en dirección noreste con una línea recta de 20.600 metros hasta el punto No. 9 de coordenadas 9880191 norte y 428433 este. Desde el punto No. 9 sigue por el cauce del Río Yasuní en dirección este hasta el punto No. 10 de coordenadas 9881333 norte y 446142 este, luego sigue en dirección este una línea recta de 6.580 metros hasta alcanzar el límite internacional en el punto No. 11 de coordenadas 9881510 norte y 453054 este.

Desde el punto No. 11 de coordenadas 9881510 norte y 453054 este, en el límite internacional, continúa una línea recta de 53.978 metros en dirección suroeste hasta alcanzar el Río Cononaco en el punto No. 12 de coordenadas 9829382 norte y 438379 este. Luego sigue el curso del Río Cononaco hasta el punto No. 13 de coordenada 9829046 norte y 435221 este. Desde el punto No. 13 en dirección suroeste se dirige una línea recta de 5.438 metros hasta el Río Curaray en el punto No. 14 de coordenada 9824846 norte y 431894 este.

Desde el punto No. 14 de coordenada 9824846 norte y 431894 este entre el límite internacional y el Río Curaray, sigue su curso hasta el punto No. 15 de coordenada 9840849 norte y 303309 este.

Desde el punto No. 15 de coordenada 9840849 norte y 303309 este en el Río Curaray sigue una línea recta de 10.190 metros en dirección norte hasta el punto No. 16 de coordenada 9851087 norte y 303347 este. Continúa otra línea recta de 10.500 metros en dirección noreste hasta el punto No. 17 de coordenada 9860130 norte y 308569 este, desde esa coordenada en dirección, norte se extiende una línea recta de 20.130 metros hasta alcanzar la coordenada del punto No. 1 inicial del límite norte de esta delimitación.

El Datum que se utiliza para la definición de los límites es: PSAD 1956 - UTM Zona 18 sur.

En esta Zona se garantizará y respetará el derecho del pueblo Huaorani y de los pueblos ancestrales en aislamiento voluntario a realizar sus tradicionales actividades de caza y pesca; así como, el uso habitual de los recursos de la biodiversidad con propósitos de subsistencias.

N° 2187

ALFREDO PALACIO GONZÁLEZ

PRESIDENTE CONSTITUCIONAL DE LA REPÚBLICA

Art. 2.- Se establece una zona de amortiguamiento de diez kilómetros de ancho contiguo a toda la zona intangible delimitada en el presente Decreto.

El objeto de la zona de amortiguamiento es establecer un área adicional de protección que, mediante la implementación de restricciones en las actividades que se desarrollen, contribuya a proteger a los grupos en aislamiento voluntario y condición de contacto inicial.

En esta zona de amortiguamiento se prohíbe la realización de actividades extractivas de productos forestales con propósitos comerciales; igualmente, se prohíbe el otorgamiento de concesiones mineras en esta zona.

Las comunidades ancestrales asentadas en la zona de amortiguamiento son las únicas que podrán realizar actividades tradicionales de caza, pesca y uso de la biodiversidad con fines de subsistencia; así como, actividades de turismo moderado y controlado, bajo un sistema de restricción y de bajo impacto. Esta actividad podrá realizarse también a lo largo del río Curaray; así como, por el río Cononaco Grande hasta el asentamiento Huaorani, conocido como Sandoval.

En el segmento de la zona de amortiguamiento ubicada al interior del Parque Nacional Yasuní, las actividades se sujetarán al Plan de Manejo de dicho parque.

Art. 3.- Se prohíbe realizar en la zona de amortiguamiento nuevas obras de infraestructura tales como carreteras, centrales hidroeléctricas, centros de facilidades petroleras; y, otras obras que los estudios técnicos y de impacto ambiental juzguen incompatibles con el objeto de la zona intangible.

El Ministerio del Ambiente será el encargado de llevar a cabo los estudios técnicos y de impacto ambiental, antes indicados.

Art. 4.- El Ministerio del Ambiente, el Ministerio de Energía y Minas y el Consejo de Desarrollo de Nacionalidades y Pueblos del Ecuador, CODENPE, en el plazo de 180 días, definirán las políticas y procedimientos adecuados para evitar o minimizar la influencia que, las actividades de las operadoras petroleras legalmente autorizadas a operar en la zona de amortiguamiento, puedan ejercer sobre la vida de los pueblos ocultos que habitan en la zona intangible.

N° 2187

ALFREDO PALACIO GONZÁLEZ

PRESIDENTE CONSTITUCIONAL DE LA REPÚBLICA

Las operaciones petroleras autorizadas deberán utilizar técnicas de bajo impacto para la exploración y explotación de hidrocarburos en la Zona de Amortiguamiento (técnicas de perforación direccionada o en racimo, tendido tubería subterránea); mismas que, tendrán que ser autorizadas por el Ministerio de Ambiente y el Ministerio de Energía y Minas.

Art. 5.- El Instituto de Patrimonio Cultural conjuntamente con el Consejo de Desarrollo de las Nacionalidades y Pueblos del Ecuador, CODENPE y el Ministerio del Ambiente, precautelarán que las actividades permitidas no afecten o incidan en las costumbres, lenguaje, manifestaciones culturales, artesanales, técnicas, artísticas, musicales, religiosas, rituales o comunitarios de los grupos ancestrales que en esta región habitan.

El Instituto de Patrimonio Cultural conjuntamente con el Consejo de Desarrollo de las Nacionalidades y Pueblos del Ecuador, CODENPE, presentarán un informe semestral al Ministerio del Ambiente, respecto del cumplimiento del objeto de la zona intangible, para avaluar y mejorar la situación de dicha zona.

Por su parte, el Ministerio del Ambiente presentará un informe anual al Presidente de la República, respecto del cumplimiento del objeto de la zona intangible.

Art. 6.- En el plazo improrrogable de ciento ochenta días, contados a partir de la vigencia del presente decreto, el Ministerio del Ambiente, con el apoyo del Ministerio de Energía y Minas y el CODENPE, realizarán la delimitación física, la que incluye la demarcación con hitos; así como la elaboración y difusión de la cartografía oficial.

Art. 7.- En el plazo de noventa días, los Ministerios de Energía y Minas, del Ambiente y de Turismo, expedirán las regulaciones para las actividades permitidas en la zona intangible y de amortiguamiento.

Las actividades de turismo moderado serán reglamentadas, en un plazo improrrogable de noventa días, por los Ministerios del Ambiente y de Turismo, y por los representantes de los pueblos Huaorani del Cononaco y las comunidades Quichua del bajo Curaray. Esta reglamentación establecerá normas obligatorias (incluyendo aspectos de bioseguridad) para operadores, guías y visitantes que garanticen la protección y respeto de los pueblos en aislamiento voluntario y condición de contacto inicial. También se establecerá un límite de visitante en base a una evaluación de la capacidad de carga.

Nº 2187

ALFREDO PALACIO GONZÁLEZ

PRESIDENTE CONSTITUCIONAL DE LA REPÚBLICA

Art. 8.- De la ejecución del presente Decreto, que entrará en vigencia a partir de la fecha de su publicación en el Registro Oficial, encárguese a los ministros de Energía y Minas, de Economía y Finanzas, de Ambiente, de Turismo y al Secretario Nacional Ejecutivo del Consejo de Desarrollo de Nacionalidades y Pueblos del Ecuador.

Dado en el Palacio Nacional, en Quito, a 3 de Enero de 2007.

ALFREDO PALACIO GONZÁLEZ
PRESIDENTE CONSTITUCIONAL DE LA REPÚBLICA

IVÁN RODRÍGUEZ RAMOS
MINISTRO DE ENERGIA Y MINAS

ANITA ALBÁN MORA
MINISTRA DEL AMBIENTE

Es fiel copia del original.- LO CERTIFICO

Dr. DIEGO REGALADO ALMEIDA
Subsecretario General de la Administración Pública
