# Supplementary material for: Uncontacted Waorani in the Yasuní Biosphere Reserve: Geographical Validation of the Zona Intangible Tagaeri Taromenane (ZITT)
Source: PLoS One. 2013 Jun 19;8(6):e66293. doi: 10.1371/journal.pone.0066293 (PMC3686793; doi:10.1371/journal.pone.0066293)
Supplement: Table S1 — Metric coordinates of the 17 given points by the Presidential Decree 2187 (2007) defining the Intangible Zone Tagaeri Taromenane. (PDF) [file pone.0066293.s008.pdf]

Table S1. Metric coordinates of the 17 given points by the Presidential Decree 2187 (2007) defining the Intangible Zone Tagaeri Taromenane.

	UTM 18s - PSAD 1956[a]		Distance to the following point (m)
No.	X (m)	Y (m)	
1	308703	9880258	9919
2	318314	9882711	4577
3	317709	9887248	5984
4	322749	9890474	13550
5	336056	9893028	3460
6	339450	9892355	23279
7	359579	9880661	50111
8	409111	9873067	20593
9	428433	9880191	17746
10	446142	9881333	6914
11	453054	9881510	54154
12	438379	9829382	3176
13	435221	9829046	5358
14	431894	9824846	129577
15	303309	9840849	10238
16	303347	9851087	10442
17	308569	9860130	20128
