# Supplementary material for: Uncontacted Waorani in the Yasuní Biosphere Reserve: Geographical Validation of the Zona Intangible Tagaeri Taromenane (ZITT)
Source: PLoS One. 2013 Jun 19;8(6):e66293. doi: 10.1371/journal.pone.0066293 (PMC3686793; doi:10.1371/journal.pone.0066293)
Supplement: Table S2 — Geospatial raster data used in GIS analyses: coverage, typology, resolution and sources. (PDF) [file pone.0066293.s009.pdf]

**Table S2. Geospatial raster data used in GIS analyses: coverage, typology, resolution and sources.**

| Coverage | Typology | Geometric resolution | Source | Year |
|---|---|---|---|---|
| Orellana, Pastaza | Digital Elevation Model (DEM) | 30 meters | IGM Ecuador | 2011 |
| Orellana, Pastaza | Digital Elevation Model (DEM) | 92 meters | SRTM | 2000 (February) |
| Orellana, Pastaza | Digital Elevation Model (DEM) | 30 meters | GDEM2 | 2009 |
| 3 satellite scenes | Landsat 5 Thematic Mapper false colour composition 4 (blue), 5 (green), 7 (red) | 30 meters | Landsat USGS | - 19th of February 1991<br>- 28th of August 2005<br>- 2nd of July 2005 |
