# Supplementary material for: Uncontacted Waorani in the Yasuní Biosphere Reserve: Geographical Validation of the Zona Intangible Tagaeri Taromenane (ZITT)
Source: PLoS One. 2013 Jun 19;8(6):e66293. doi: 10.1371/journal.pone.0066293 (PMC3686793; doi:10.1371/journal.pone.0066293)
Supplement: Table S3 — Characteristics and sources vector geodata. (PDF) [file pone.0066293.s010.pdf]

Table S3. Characteristics and sources vector geodata.

Geographical layers	Geometrical structure	format	Source	year
**River network**	line	shape	Geo-portal IGM Ecuador	2012
**River basin**	polygon	shape	ECORAE	2008
**Urban centers**	point	shape	ECORAE	2008
**Road network**	line	shape	ECORAE	2008
**New roads**	line	shape	GPS survey, Pappalardo (Thesis, QD)	2009 2010 2011
**Local communities**	point	shape	ECORAE	2008
**Local communities**	point	shape	GPS survey, (Pappalardo)	2009 2012
**Tagaeri Taromenane clans distribution**	polygon	shape	Ministry of Justice (PMC)	2012
**Yasuní National Park**	polygon	shape	World Database of Protected Areas	2010
**Waorani Indigenous Reserve**	polygon	shape	World Database of Protected Areas	2009
**Intangible Zone Tagaeri Taromenane (ZITT)**	polygon	shape	Justice Ministry (PMC)	2011
**Oil blocks**[a]	polygon	shape	Secretaria de Hidrocarburos de Ecuador	2012
**Oil fields**	polygon	shape	ECORAE	2008
**Oil wells (exploratory, productive and injectors)**	points	shape	ECORAE	2008
